# HSC Niche Dynamics in Regeneration, Pre-malignancy, and Cancer: Insights From Mathematical Modeling

**DOI:** 10.1093/stmcls/sxac079

**Published:** 2022-11-13

**Authors:** Rasmus Kristoffer Pedersen, Morten Andersen, Vibe Skov, Lasse Kjær, Hans C Hasselbalch, Johnny T Ottesen, Thomas Stiehl

**Affiliations:** IMFUFA, Department of Science and Environment, Roskilde University, Roskilde, Denmark; Centre for Mathematical Modeling - Human Health and Disease, Roskilde University, Roskilde, Denmark; IMFUFA, Department of Science and Environment, Roskilde University, Roskilde, Denmark; Centre for Mathematical Modeling - Human Health and Disease, Roskilde University, Roskilde, Denmark; Department of Hematology, Zealand University Hospital, Roskilde, Denmark; Department of Hematology, Zealand University Hospital, Roskilde, Denmark; Department of Hematology, Zealand University Hospital, Roskilde, Denmark; IMFUFA, Department of Science and Environment, Roskilde University, Roskilde, Denmark; Centre for Mathematical Modeling - Human Health and Disease, Roskilde University, Roskilde, Denmark; IMFUFA, Department of Science and Environment, Roskilde University, Roskilde, Denmark; Centre for Mathematical Modeling - Human Health and Disease, Roskilde University, Roskilde, Denmark; Institute for Computational Biomedicine - Disease Modeling, RWTH Aachen University, Aachen, Germany

**Keywords:** mathematical modeling, stem cell niche, clonal competition, myeloid malignancies, stem cell fitness

## Abstract

The hematopoietic stem cell (HSC) niche is a crucial driver of regeneration and malignancy. Its interaction with hematopoietic and malignant stem cells is highly complex and direct experimental observations are challenging. We here develop a mathematical model which helps relate processes in the niche to measurable changes of stem and non-stem cell counts. HSC attached to the niche are assumed to be quiescent. After detachment HSC become activated and divide or differentiate. To maintain their stemness, the progeny originating from division must reattach to the niche. We use mouse data from literature to parametrize the model. By combining mathematical analysis and computer simulations, we systematically investigate the impact of stem cell proliferation, differentiation, niche attachment, and detachment on clinically relevant scenarios. These include bone marrow transplantation, clonal competition, and eradication of malignant cells. According to our model, sampling of blood or bulk marrow provides only limited information about cellular interactions in the niche and the clonal composition of the stem cell population. Furthermore, we investigate how interference with processes in the stem cell niche could help to increase the effect of low-dose chemotherapy or to improve the homing of genetically engineered cells.

## Graphical Abstract

**Figure:**
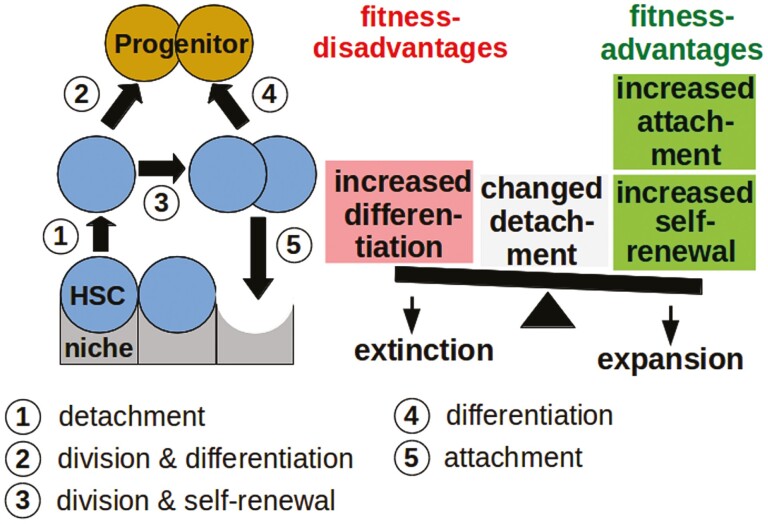
Processes in the hematopoietic stem cell (HSC) niche impact clonal fitness. Increased self-renewal or attachment rates confer a competitive advantage to a mutated clone, increased differentiation implies a disadvantage. Detachment rates have no impact. Depending on these processes the mutated cell burden in the stem cell niche can be higher or lower compared to progenitors and mature cells.

**Significance Statement**

The hematopoietic stem cell (HSC) niche is involved in progression of myeloid malignancies, therapy-induced malignant cell eradication, HSC mobilization, and engraftment of genetically engineered cells. We present a mathematical modeling approach for studying the interactions between stem cells and niche cells (attachment, detachment, competition) and for simulating novel treatment ideas. This allows for quantification of HSC fitness based on interactions within the HSC niche. Simulations imply that sampling of blood, whole marrow, or primitive progenitors may not reflect the cellular composition of the stem cell compartment and thus not correlate with disease progression or therapy outcome.

## Introduction

Adult stem cells drive tissue maintenance and regeneration. A paradigmatic example is the blood-forming (hematopoietic) system. In a multi-step process, hematopoietic stem cells (HSC) give rise to all types of mature blood cells.^1^ To preserve their functionality, HSC require complex interactions with their micro-environment, the so-called stem cell niche.^1^

Similar to adult stem cells, cancer stem cells (cancer initiating cells) give rise to a heterogeneous malignant cell bulk and are required for persistence and expansion of the cancer. Key diseases of the hematopoietic system, such as chronic myeloid leukemia (CML), acute myeloid leukemia (AML), or the Philadelphia-chromosome negative myeloproliferative neoplasms (MPN), are triggered by cancer stem cells (CSC).^2-5^ Animal models and clinical data support the concept that CSC impair the stem cell niche and out-compete HSC.^5-8^ A similar mechanism underlies clonal hematopoiesis of indeterminate potential (CHIP) where mutated stem and progenitor cell clones expand over time.^9^

Due to its involvement in various diseases, the stem cell niche is the target of several clinical interventions.^10^ Cancer therapies attempt to eradicate CSC from the niche. Agents which detach stem cells from the niche are used for HSC mobilization.^11^ HSC transplantation (HSCT)^11^ and HSC-based gene therapy^12^ aim at sustainable colonization of the niche by donor-related or genetically engineered stem cell clones.

Stem cell dynamics also play a role in diagnostics and follow-up of malignant diseases. Minimal residual disease (MRD) is a well-established approach to monitor treatment response and progression of different hematological cancers.^13^ In practice, MRD is quantified using peripheral blood or unsorted marrow samples. To assess if such samples can provide reliable information about the CSC burden, detailed insights in niche-mediated processes and their relation to the time evolution of precursor, progenitor, and mature cell counts are required.^5-7,9^

Experimental approaches which have substantially contributed to our knowledge of the HSC niche are coculture studies,^10^ transplantation experiments,^5^ time-lapse imaging,^14^ and cell-tracking.^15,16^ However, ethical and technical issues, especially the current inability to expand human HSC *in vitro*,^10^ limit our understanding of human HSC dynamics. Mechanistic mathematical and computational models can help close this gap, as they relate processes in the HSC niche to measurable quantities such as mature blood cell counts or disease progression.^6-8,17-24^

Mechanistic modeling is a potent approach for studying the properties of complex systems and to select the best fitting out of multiple competing hypotheses. It has a long tradition in hematology^25,26^ and has led to novel quantitative information about stem cell dynamics in health^8,18,19,22,27,28^ and disease.^6-8,20,21,23-25,29-43^

Here, we present a novel mechanistic mathematical model of the HSC niche. The model is parameterized based on the results of mouse experiments described in literature and its dynamics are related to clinical observations. It accounts for HSC detachment from the niche, attachment to the niche as well as stem cell proliferation, self-renewal, and differentiation. The focus of this study is to understand how these processes affect the time evolution of healthy and cancerous cell populations. We use the model to address the following specific questions, which relate closely to the clinical aspects mentioned above:

1. What are the prerequisites for the long-term persistence of a stem cell population in the niche? Which mechanisms mediate the fitness of a stem cell clone?
2. What determines if two or more stem cell clones can coexist in the niche?
3. Which therapeutic approaches facilitate the eradication of stem cell clones from the niche?
4. Can we use peripheral blood or bulk marrow samples to infer the dominant clones in the stem cell niche?
5. How can donor-derived or genetically engineered stem cell clones be introduced to the niche and persist for long times?

Our study reveals a complex impact of processes in the HSC niche on cell population dynamics. We discuss potential pitfalls related to the intuitive interpretation of clinical or experimental data.

Specifically, our model suggests that the abundance of a specific mutation (or cell clone) in the peripheral blood and in the marrow cell bulk may considerably differ from the abundance of the respective mutation (or clone) in the stem cell population. This may impact the interpretation and clinical use of measurements obtained from peripheral blood or unsorted marrow samples.

Based on model analysis and simulation, we identify which stem cell properties imply selective advantages. Moreover, we ask whether low-dose preconditioning combined with mobilizing agents and HSCT could serve as an alternative to conventional AML therapy in patients with multi-morbidity.

## Methods

### Mathematical Modeling

**Figure 1.**
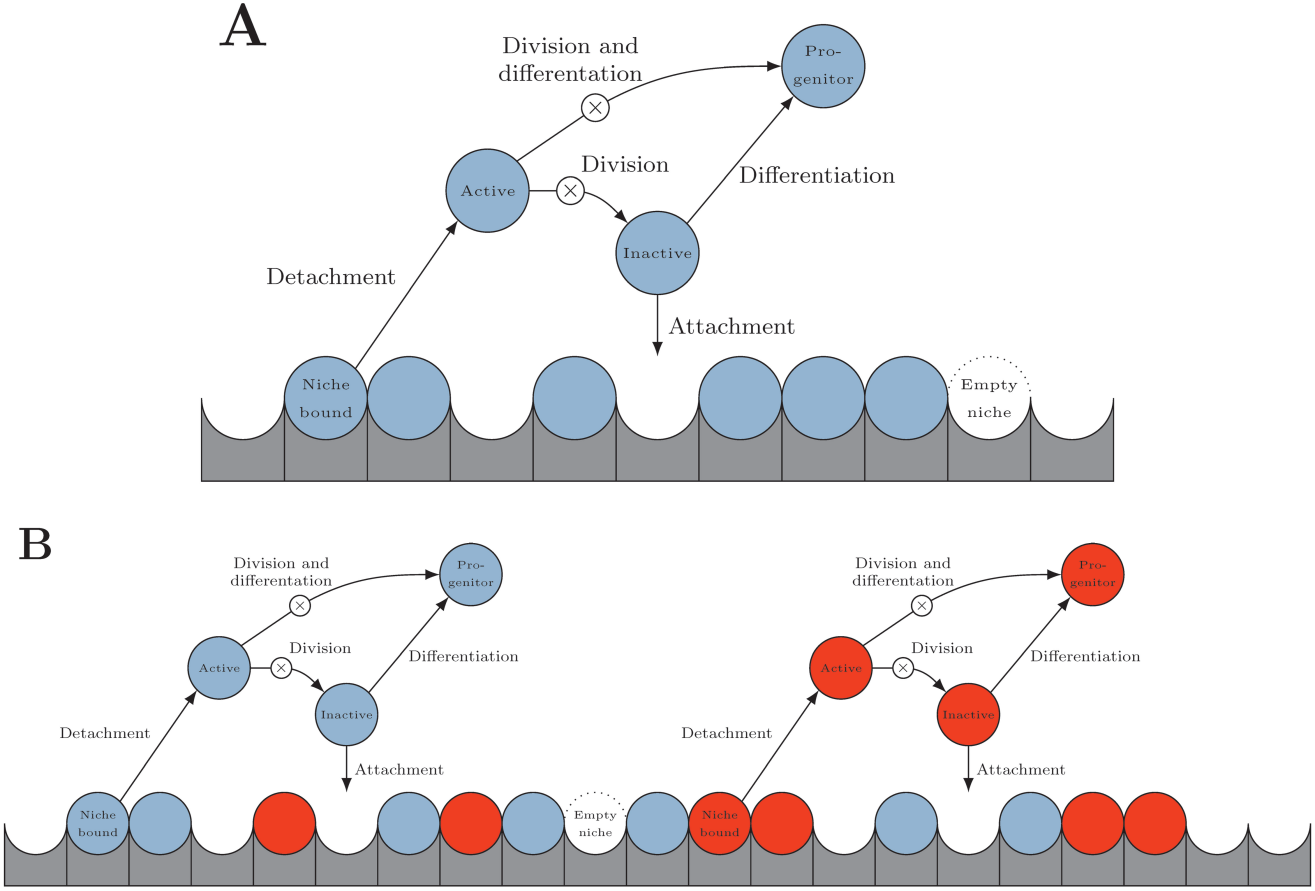
Illustration of the mathematical model. Stem cells are depicted as light blue and dark red circles (for colour figure refer to online version). Stem cell niches are illustrated by gray blocks. Panel (**a**) shows a scenario with a homogeneous stem cell population. Panel (**b**) illustrates a scenario with 2 stem cell clones competing for spaces in the joined niche. Arrows illustrate the processes considered in the model: Detachment from the niche, attachment to an empty niche space, self-renewing division and differentiation.

The proposed model accounts for interactions of stem cells with the HSC niche. It allows us to infer how these interactions impact blood cell formation and progression of stem cell-driven diseases. The model is a 6-dimensional system of ordinary differential equations with 11 non-negative parameters. The model is illustrated in Fig. 1. A detailed description is provided in Supplementary Section S1 and Fig. S1.

We assume that the capacity of the HSC niche is finite and constant in time.^44^ The capacity is defined as the maximal number of stem cells that can reside in the niche. In the model, stem cells detach from the niche with a constant probability per unit of time. During detachment stem cells get activated.^11,45-47^ Activated HSC either divide or differentiate. Upon division stem cells produce progeny which are again stem cells, upon differentiation stem cells irreversibly adopt the progenitor fate, whereas stem cells can self-renew indefinitely, progenitors have a reduced self-renewal potential and further differentiate after a finite number of divisions.

We assume that upon division the stem cells enter a state which we denote as inactive. The inactive cells either reattach to the niche or differentiate. The inactive state is required to reproduce the observation that stem cells decline in absence of the niche, see Supplementary Section S1.4. and Supplementary Fig. S2. As such this state is distinct from cellular quiescence. Hence, we consider 3 states: active, inactive, and quiescent. Activation of stem cells from quiescence^19–22,25^ and the mechanisms underlying loss of stemness^6-8,18,20,22,48-51^ have been modeled extensively in the literature. Our distinction between different states of non-quiescent stem cells, ie, active and inactive, is new and has been introduced as a hypothetical mechanism behind the observation that stem cells cannot persist in absence of an appropriate niche.^10^ A similar mechanism has been considered previously in the literature by introducing a niche affinity which decreases in dividing cells.^22^ Pioneering modeling works accounting for the transition of HSC between active and resting states are the works of Mackey^25^ and of Rubinow and Lebowitz.^52^

The following parameters quantify the processes accounted for by our model. The detachment rate is defined as the proportion of niche-bound stem cells detaching per unit of time. The attachment rate determines which fraction of unbound stem cells attaches to the niche per unit of time. The division frequency of active stem cells is quantified by the proliferation rate, the differentiation of stem cells per unit of time is determined by the differentiation rate. In summary, our model considers 3 different stem cell subpopulations, namely, active stem cells (not attached to the niche), inactive stem cells (not attached to the niche) originating from divisions of active stem cells and quiescent stem cells (attached to the niche). Furthermore, it accounts for empty niche spaces. We used published data from mouse experiments to fit the parameters of HSC. This was accomplished using unbounded simulated annealing, in which 5 pairs of parameters are assumed to be pairwise equal. As cost function we used the sum of squared residuals. Details are provided in Supplementary Section S2 and Figs. S3-S4. Note that model parameters are not uniquely identifiable from the data considered; however, model dynamics were found to be comparable for other choices of parameter values. A mathematical analysis of a class of related models is available.^53^

The parameterized model has a positive equilibrium corresponding to healthy homeostasis and a trivial equilibrium corresponding to death of the organism. In the absence of the stem cell niche, HSC are lost due to differentiation (Supplementary Fig. S5), as observed in experiments.^10^ When the cell counts of the homeostatic state are perturbed, eg, due to blood loss, the system recovers and the healthy equilibrium is re-established (Supplementary Fig. S6). Corresponding details and mathematical analysis are provided in Supplementary Sections S3 and S4.

To study clonal evolution, we proposed an extension of the model that accounts for 2 stem cell clones (Fig. 1B). Per definition, stem cells with identical genomes belong to the same clone. Therefore, stem cells from the same clone have identical cell properties, ie, can be characterized by the same set of model parameters. We do not explicitly consider the accumulation of mutations which is linked to rare and random events. Mutations are accounted for in terms of cell properties: mutated cells have different proliferation, attachment, detachment rates, etc., compared with wild-type cells, which can confer a growth advantage or disadvantage. This approach is widely used.^7,8,32-34,39,52^

## Results

### The Homeostatic Number of Circulating HSC Increases for Increased HSC Attachment Rate

In homeostasis stem cell attachment, detachment differentiation and proliferation are in a dynamic equilibrium. We used our model to assess how the homeostatic cell counts depend on each of these processes (Table 1; Supplementary Figs. S7, S8). Some of the observed effects are intuitive: increased proliferation of stem cells is linked to increased homeostatic stem cell counts inside and outside the niche. In response to an increase of the differentiation rate, homeostatic stem cell counts decline. In agreement with experiments,^44^ an increased niche capacity results in higher equilibrium stem cell counts.

**Table 1.** Summary of the effect of parameter changes on equilibrium cell counts.

Increased parameter	Niche-bound *N*_H_	Inactive *I*_H_	Active *A*_H_	Progenitors *i*_DH_
Niche capacity, *K*	↑	↑	↑	↑
Detachment from niche, *u*	0	↑	↑	↑
Division rate, *r*	↑	↑	*	↑
Differentiation of active, *d*_A_	↓	↓	↓	↓
Differentiation of inactive, *d*_I_	↓	↓	↓	↓
Attachment to the niche, *b*	↑	↑	↑	↑

↑ indicates that the value of the respective variable increases if the value of the respective parameter is increased, ↓ indicates that the value of the respective variable decreases if the value of the respective parameter is increased. *Increasing only if division rate of active stem cells, *r*, is close to the differentiation rate of active stem cells, *d*_A,_ otherwise decreasing, see Supplementary Fig. S8.

As in mouse experiments,^54^ the model exhibits empty niche spaces in physiological homeostasis. Furthermore, in agreement with data from healthy individuals, a certain proportion of HSC is detached from the niche under homeostatic conditions.^55,56^

The number of empty niche spaces in homeostasis equals $d_{I}\;\cdot \;\left(r+d_{A}\right)/(b\;\cdot \;\left(r-d_{A}\right))$, where *d*_*I*_ and *d*_*A*_ denote the differentiation rates of inactive and active cells, *b* is the rate at which cells attach to the niche, and *r* is the division rate of active cells. High differentiation rates result in a high number of empty niches in equilibrium, high attachment and proliferation rates result in a small number of empty niches in equilibrium. For the parameters considered 0.4% of the niche spaces are empty. The equilibrium ratio of inactive and active stem cells is independent of the attachment and detachment rates and given by (*r* − *d*_*A*_)/*d*_*I*_ (Supplementary Sections S4.1, S4.2).

**Figure 2.**
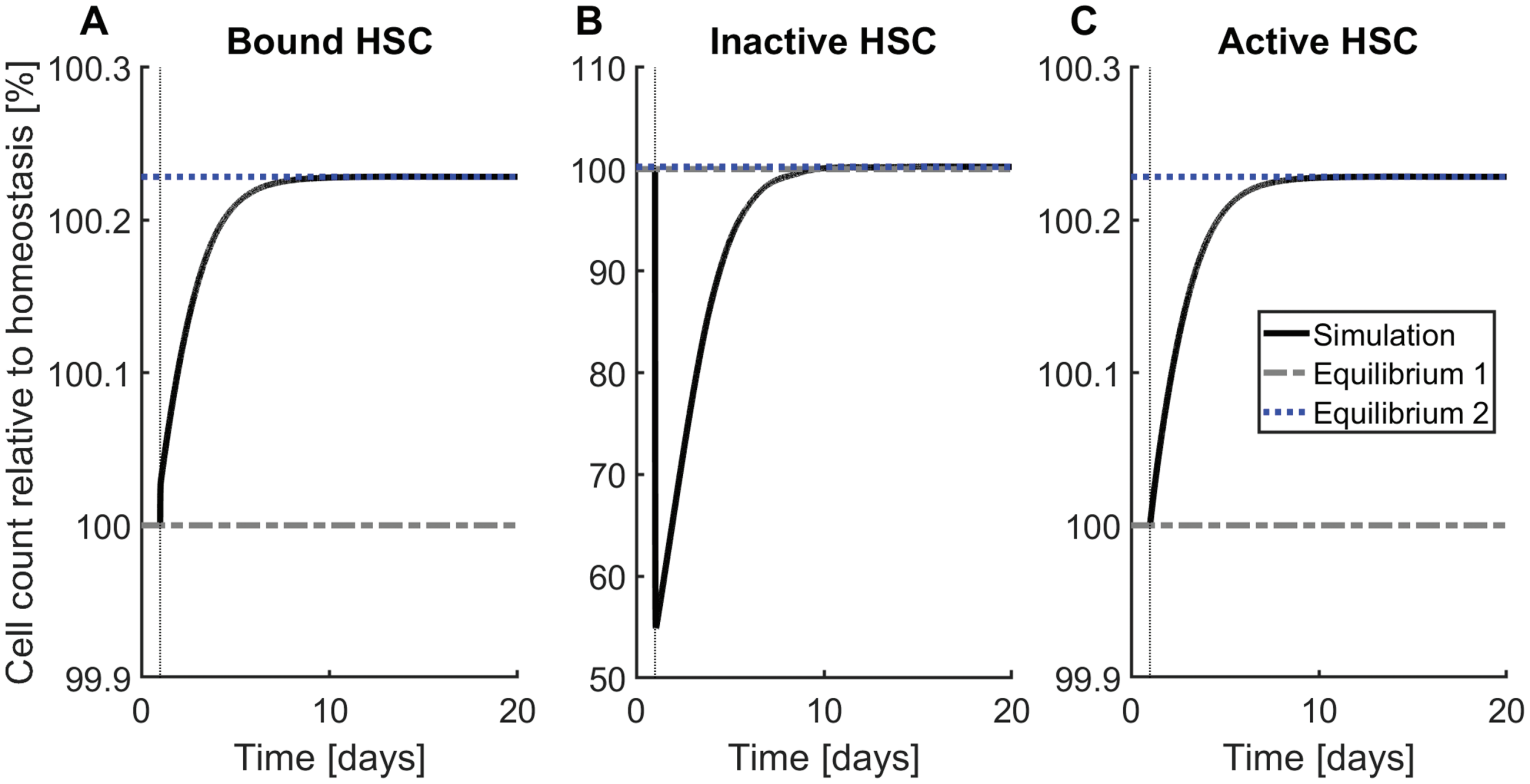
System dynamics in response to changes in HSC attachment rate. Starting at equilibrium, the HSC attachment rate is doubled from day 1 until the end of the simulation. The system converges to a new equilibrium, with both higher niche-bound HSC counts (**A**) and higher free (active and inactive) cell counts (**B**,**C**). Equilibrium 1 refers to the homeostatic state and Equilibrium 2 refers to the equilibrium reached after the attachment rate has been increased. The observations can be explained as follows: A higher attachment rate leads to an increased binding of free cells to the niche and a consecutive temporal decrease of unbound HSC. However, the higher HSC number in the niche leads to an increase in the number of HSC detaching (and thereby getting activated) per unit of time and thus, on a longer time scale, to an increase of free cells. The simulation suggests that the homeostatic HSC number is relatively insensitive to the attachment rate. Assuming a stem cell count of approximately 10^4^ in humans,^68^ a change of the depicted magnitude corresponds to approximately 20 bound HSC.

Model simulations show that an increased rate of stem cell attachment results in higher homeostatic stem cell counts inside and outside the bone marrow niche (Fig. 2). This is a result of the following mechanism: Intuitively, a persistent increase of the attachment rate is linked to an increase of attached stem cells. Temporarily, the number of unbound HSC decreases in response to the increased attachment. However, as long as the detachment rate remains constant, the number of stem cells detaching per unit of time (and thereby getting activated for division) increases for increasing numbers of attached cells. This explains why the number of unbound stem cells grows after an initial decline.

### The Equilibrium Count of HSC in the Niche Cannot Be Inferred Solely From Circulating HSC Counts

**Figure 3.**
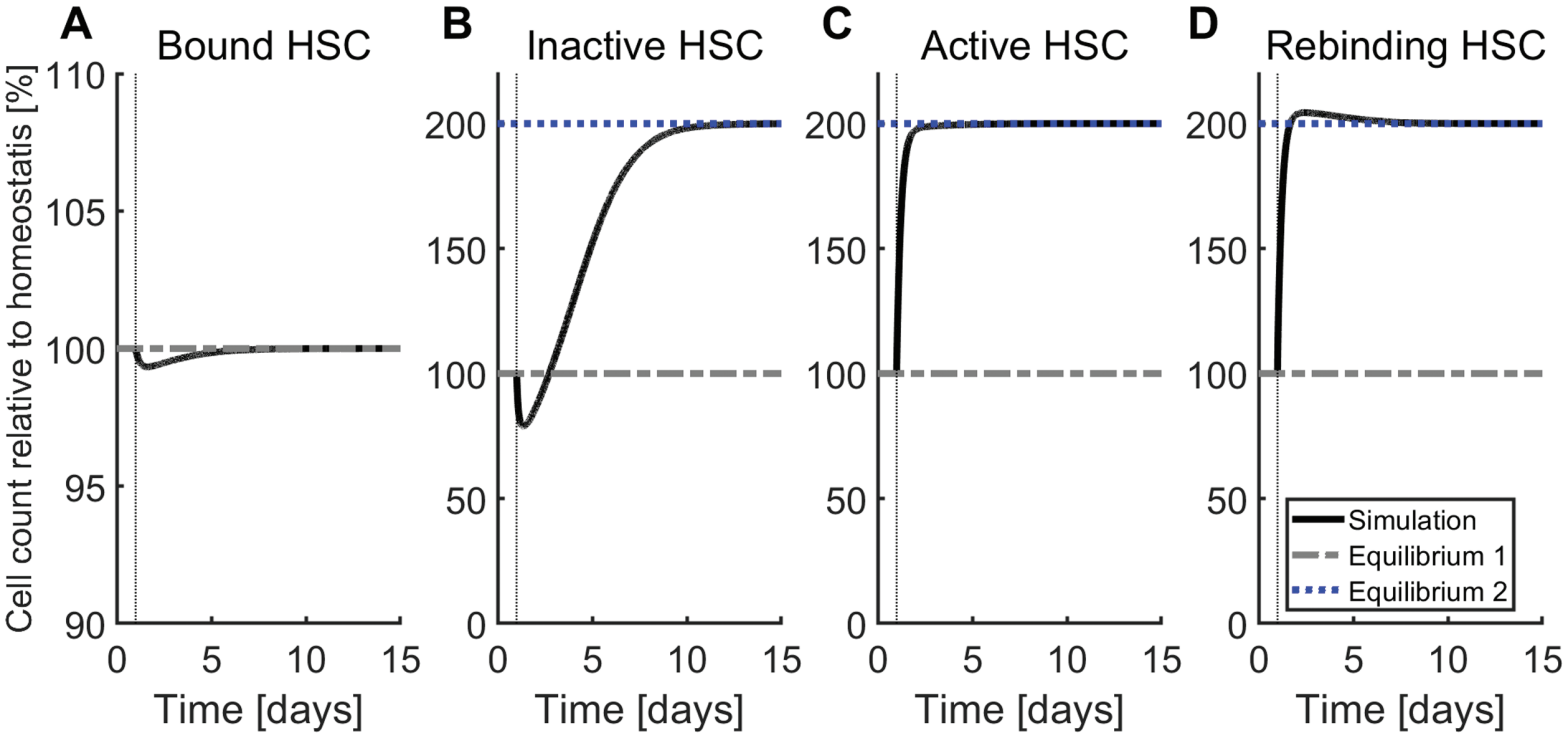
System dynamics in response to changes in HSC detachment rate. Starting at equilibrium, the detachment rate is doubled from day 1 until the end of the simulation. The system converges to a new equilibrium with an unchanged number of niche-bound HSC (**A**) and with both higher inactive (**B**) and active HSC (**C**). Equilibrium 1 refers to the homeostatic state and Equilibrium 2 refers to the equilibrium reached after perturbation of parameter values. (**D**) The amount of free stem cells binding to the niche per unit of time.

Model analysis implies that the homeostatic number of empty niches is independent of the HSC detachment rate (Fig. 3). The mechanism underlying this observation is as follows: Since HSC become activated upon detachment, increased detachment rates result in an increased number of proliferating HSC and hence in increasing HSC counts outside the niche. The latter leads to an increased number of HSC which attach to the niche and thus compensate for the detaching cells. Comparing Fig. 2 with Fig. 3 implies that different scenarios can result in very similar long-term dynamics. This results from the interplay of the different processes in the niche where perturbation of one process can be counterbalanced by other processes. Therefore, the homeostatic counts of unbound or circulating HSC cannot be used to infer how many stem cells are attached to the niche. According to Figs. 2-3, a raise of unbound HSC in homeostasis can occur in response to both increased attachment and increased detachment rates. Although both changes have the same effect on the homeostatic number of unbound stem cells, they have different effects on the number of attached stem cells.

### Increased Stem Cell Differentiation Results in Reduced Homeostatic Progenitor Production

Since non-stem cells can divide only a finite number of times, the mature cell production scales with the production of progenitors. The latter corresponds to the number of stem cells that differentiate into progenitors per unit of time. For this reason, conditions which increase the number of stem cells in homeostasis also increase the progenitor production in the model. Examples for this are increased niche capacity or increased proliferation rate (Table 1; Supplementary Fig. S7, Sections S4.2-S4.3). Since it leads to a raise of proliferating HSC,^45^ an increase of the HSC detachment rate is also linked to an increased progenitor production.

**Figure 4.**
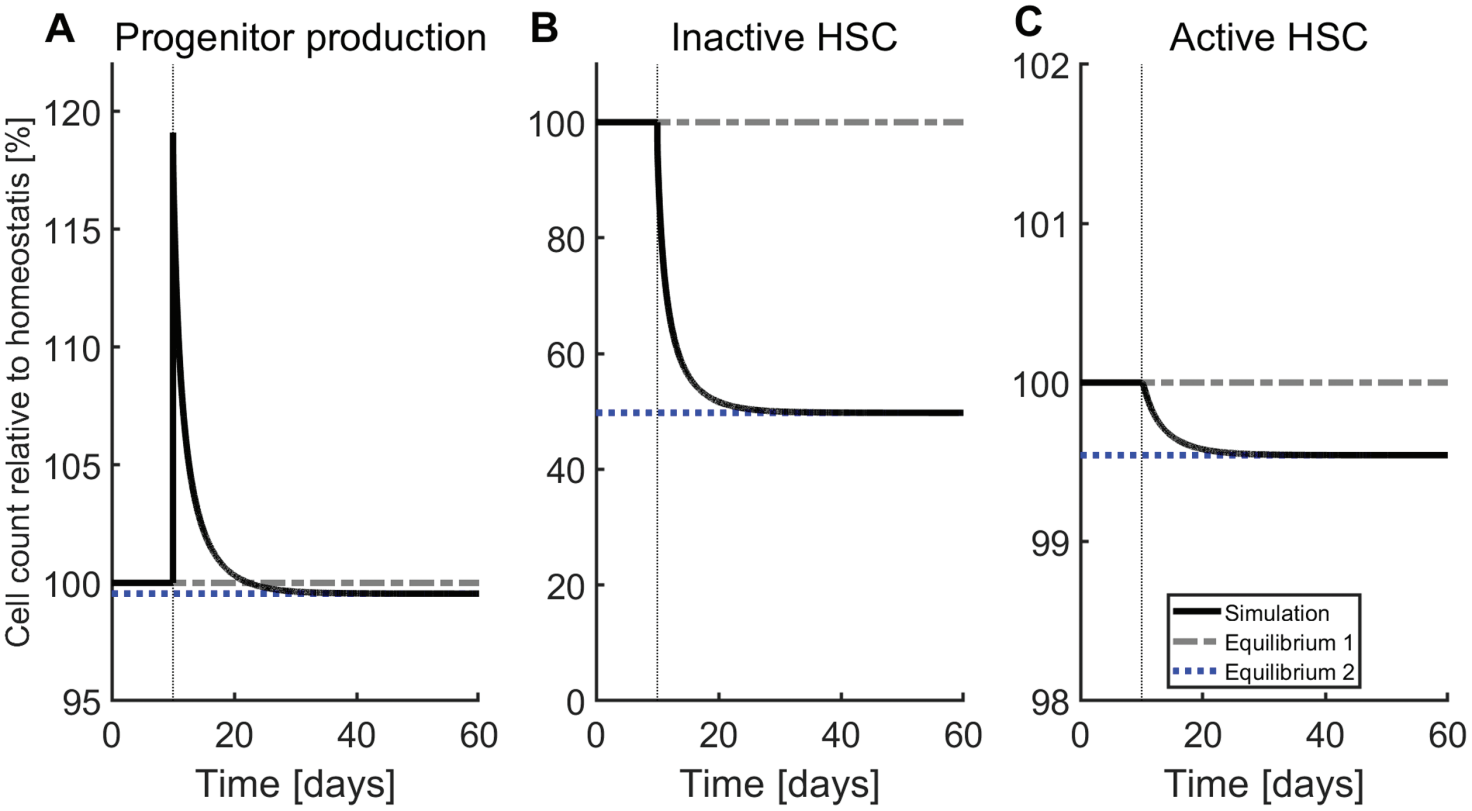
Production of progenitors following changes of the differentiation rate. Starting at equilibrium, the differentiation rate of inactive HSC is doubled from day 10 until the end of the simulation. A temporary increase in the production of progenitors is seen (**A**), however, after some time a new homeostatic equilibrium with reduced progenitor outputs is approached. The stem cells converge to a new equilibrium, with both lower inactive (**B**) and active (**C**) HSC outside the niche.

In case of a persistent increase of HSC differentiation rate, homeostatic progenitor production decreases (Fig. 4). Transiently the output of progenitors increases, however, since an increased differentiation leads to a decline of HSC, progenitor production eventually decreases. These results coincide with previous findings.^48^

### Niche-Binding, Differentiation and Proliferation Rates Determine the Fitness of a Stem Cell Clone

Stem cell-driven malignant or pre-malignant diseases result from the growth of a mutated stem cell clone which interferes with healthy cells.^4-7^ Systematic model analysis enables us to identify which cell properties confer a competitive advantage to a stem cell clone. Assuming that disease-related genetic, epigenetic, or metabolic alterations impact cell properties, this approach provides insights into disease dynamics. According to our model, competitive advantages result from reduced differentiation, increased proliferation, or increased binding to the niche. This is in line with previous results.^6-8,20,32,33,53^

Since disadvantageous alterations of a specific cell trait can be compensated by advantageous alterations of other traits, it is a complex task to predict whether certain cell properties imply a growth advantage compared with the wild type or not. For this reason, we derived a mathematical expression that quantifies the fitness of a clone. We define the fitness of a clone as its ability to out-compete wild-type cells or other clones from the niche. The fitness is calculated as


$$F_{H}=\frac{b_{H}}{d_{I_{H}}}\frac{\left(r_{H}-d_{A_{H}}\right)}{\left(r_{H}+d_{A_{H}}\right)}.$$

Here, $b_{H}$ is the binding rate, $d_{A_{H}}$ is the differentiation rate of active stem cells, $d_{I_{H}}$ is the differentiation rate of inactive stem cells, and $r_{H}$ is the proliferation rate of active stem cells. A clone can only persist in the niche if the stem cell gain due to proliferation compensates or outweighs the stem cell loss due to differentiation, ie, if $r_{H}>d_{A_{H}}$. If 2 clones compete for the niche, the clone with the higher fitness value out-competes the clone with the lower fitness value. The expression for the fitness emerges from the mathematical analysis of our nonlinear model (Supplementary Section S4.4, Fig. S9). If the stem cell binding rate $b_{H}$ increases, the fitness *F*_H_ increases. This is in line with the intuition that the stronger the binding of a cell to the niche, the more difficult it is to out-compete the cell from the niche. Similarly, if the proliferation rate $r_{H}$ increases, the fitness *F*_H_ increases. This is intuitive, since the faster stem cells produce offspring, the more difficult it is to drive their population to extinction. If the differentiation rates $d_{I_{H}}$ or $d_{A_{H}}$ increase, the value of *F*_H_ decreases, since the more stem cells are lost per unit of time due to differentiation, the slower the growth of the stem cell population and the easier it is out-competed by a fast growing clone. The term (*r*_*H*_ − *d*_*A*__*H*_) is proportional to the number of progeny stem cells arising from activated HSC. The term (*r*_*H*_ + *d*_*A*__*H*_) is proportional to the loss of active stem cells per unit of time due to differentiation or inactivation. The term *b*_*H*_/*d*_*I*__*H*_ quantifies which fraction of the inactive stem cells is lost due to differentiation. Therefore, the interpretation of the formula is intuitive: The more the production of new stem cells outweighs the loss of stem cells due to differentiation, the higher is the fitness of the clone.

The formula quantifies how an advantage in one property can counterbalance a disadvantage in other properties. For example, if the differentiation rate $d_{I_{H}}$ is doubled, and at the same time the binding rate $b_{H}$ is doubled, the fitness of the clone remains unchanged. In case of a manifest leukemia, the leukemic cells’ fitness $F_{L}=\frac{b_{L}}{d_{I_{L}}}\frac{\left(r_{L}-d_{A_{L}}\right)}{\left(r_{L}+d_{A_{L}}\right)}$ is larger than the fitness $F_{H}=\frac{b_{H}}{d_{I_{H}}}\frac{\left(r_{H}-d_{A_{H}}\right)}{\left(r_{H}+d_{A_{H}}\right)}$ of the HSC.

Whenever 2 clones differ with respect to their fitness, the clone with superior fitness will eventually out-compete the clone with inferior fitness. However, it depends on the specific parameters how long it takes for the inferior clone to decline and whether this happens within the life-time of the host. A scenario where the fitness of all clones is equal corresponds to neutral competition.

**Figure 5.**
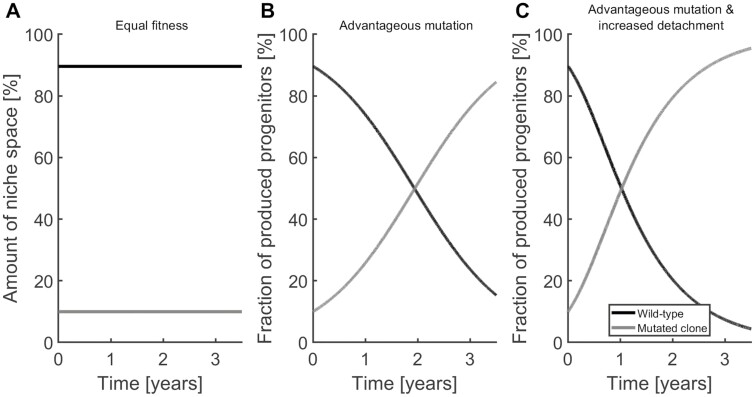
Increased detachment rate of the fitter clone leads to faster extinction of the inferior clone. In all 3 panels, 10% of the niche-bound stem cells belong to the mutated clone at the beginning of the simulations. In panel **A**, the 2 subpopulations have equal fitness; therefore, the cell counts do not change over time. In panel **B**, the division rate of the mutated clone is increased by 20% compared with the healthy clone. This results in a competitive advantage and eventual out-competition of the healthy cells. In panel **C**, the detachment rate of the mutated clone is tripled in addition to the increased division-rate. The increased detachment leads to an increased activation and thus to faster out-competition of the healthy cells.

The fitness is independent of the detachment rate. Therefore, mobilization of malignant cells from the niche is not sufficient to eradicate a disease. However, if a given clone already has a competitive advantage, an increase of its detachment rate accelerates the decline of the inferior clone (Fig. 5).

### Clonal Fitness Can Be Quantified by the Abundance of Vacant Niches in Monoclonal Homeostasis

Mathematical analysis reveals an interesting relation between the fitness of a clone and the number of vacant niche spaces. In a setting where a single stem cell clone populates the niche, the homeostatic number of empty niche spaces is given by 1/F, where F denotes the clonal fitness defined above. Consequently, the more empty niche spaces exist in a monoclonal equilibrium, the lower the clonal fitness of the respective clone. The derivation of this result is presented in Supplementary Section S4. It suggests that the fitness of a clone can be determined in absence of competition by quantifying the empty niche spaces. Possibly, *in vitro* niches or xeno-transplantation models could be used to determine clonal fitness and to predict the dynamics of clonal competition.

### The Malignant Cell Burden in Blood or Bulk Marrow Samples May Differ From the Malignant Cell Burden in the Niche

**Figure 6.**
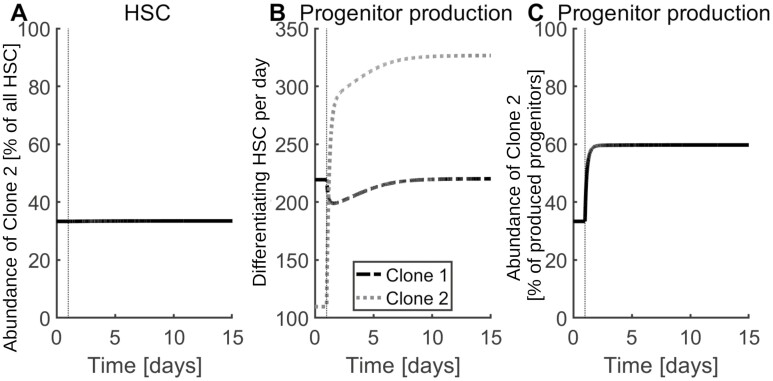
Impact of stem cell detachment rate on clonal abundance in the niche and the progenitor population. The figure considers the coexistence of 2 HSC clones with equal fitness (clone 1 and clone 2). The abundance of clone 2 in the stem cell population is defined as the percentage of stem cells derived from clone 2 among all stem cells. The abundance of clone 2 among the produced progenitors is defined as the percentage of progenitors derived from clone 2 among all produced progenitors. To study how the clonal abundance changes if the HSC detachment rate is perturbed, the detachment rate of HSC derived from clone 2 is tripled from *t* = 1 until the end of the simulation. This does not impact its fitness, as argued in section “*Niche-binding, differentiation and proliferation rates determine the fitness of a stem cell clone*”. In reaction to this change the abundance of clone 2 in the stem cell niche does not change (**A**). However, the absolute progenitor production by clone 2 increases (**B**) as well as the abundance of clone 2 in the progenitor population (**C**). This demonstrates that the majority of progenitors (and hence circulating cells) can be derived from the smaller stem cell clone. Since the contribution of a clone to the progenitor cell output correlates with the contribution of the clone to the peripheral blood cell population, it follows that abundance of a clone in the peripheral blood not necessarily reflects the abundance of the respective clone in the stem cell compartment.

The niche is involved in regulation of quiescence and proliferation.^45^ In the model, stem cells divide after detachment from the niche, as experimentally observed.^45^ The progeny originating after division differentiate or reattach to the niche. Computer simulations indicate that the malignant cell burden in the stem cell niche may differ considerably from the malignant cell burden in more mature cell fractions (progenitors, precursors, mature cells; Fig. 6, and Supporting Information Section S5, Supplementary Fig. S10). Consequently, MRD assessment based on blood or unsorted marrow samples may not reflect the frequency of CSC in the stem cell niche. Similarly, the abundance of donor-derived stem cells in the niche may differ from the chimerism measured in peripheral blood after allogeneic HSCT. Analogous conclusions hold for the chimerism or MRD in the progenitor and precursor cells populations.

The reason for this observation is that different clones may contribute differently to the production of progenitors and mature cells. If stem cells of a mutated clone mostly give rise to stem cells and only rarely produce progenitors, the abundance of the respective clone in the niche is much higher compared with its abundance in the progenitor fraction and vice versa. As discussed above, the clonal fitness is independent of the stem cell detachment rate. This implies that clones with equal fitness can differ considerably in terms of their detachment rates which implies that they give rise to different amounts of progenitors per unit of time. This reasoning applies to scenarios with non-neutral (Supplementary Fig. S10) and neutral competition (Fig. 6).

One example for neutral competition is a mutant clone with increased detachment but unchanged proliferation, attachment and differentiation rates compared with the wild-type HSC. Such a clone gives rise to large amounts of circulating cells but cannot out-compete wild-type HSC (Fig. 6). In individuals with CHIP, the allele frequency of *JAK2V617F* or *CALR* is analyzed in peripheral blood or bulk marrow.^57,58^ Our model implies that samples enriched for more immature cells might be more suitable to infer the allele frequencies in the stem cell population. Another consequence of this finding is that mutations which increase the number of progenitor or mature cells generated by a certain clone not necessarily increase its competitive advantage.

### Unpreconditioned Patients Could Benefit From a Combination of Mobilizing Agents and Multi-Dose HSCT

Cellular interactions in the bone marrow niche are closely intertwined with engraftment dynamics after HSCT. Xenotransplantation experiments in unpreconditioned mice reveal that the number of donor-derived cells increases if the graft is divided into multiple portions transplanted at subsequent days.^54^ Dynamic simulations of our model are in line with this observation (Supplementary Section S6; Supplementary Fig. S4). According to our model, this effect decreases if the dose of preconditioning increases (Supplementary Fig. S11). This coincides with a clinical trial reporting no benefit of multi-dose transplantation after high dose preconditioning.^59^ The advantage of multiple transplant doses is linked to the dynamic detachment and attachment of HSC to the niche. In the time interval between the transplantations some HSC detach from the niche, a certain fraction among them is host derived. These HSC give rise to empty niches, which, upon transplantation of the next dose of donor-derived cells, are mostly filled by donor-derived HSC. Thus, the fraction of donor-derived HSC increases with every transplant dose. Setting the detachment rates of host-derived HSC to zero the advantage of multiple transplant doses can no longer be observed in model simulations.

Stem cell mobilizing agents can perturb processes in the niche.^46,47^ Our model helps to hypothesize under which conditions such agents might improve the outcome of medical interventions and under which conditions they might have adverse effects. Especially in multi-morbid patients conventional AML chemotherapy is a limiting factor. For this reason, the question arises whether myeloablation can be partially replaced by mobilization protocols which force stem cells to leave the niche. We simulated the outcome of a therapeutic regimen consisting of mobilizing agents followed by HSCT. According to our model, this approach might reduce the CSC burden. Nevertheless, compared with an HSCT without prior mobilization the reduction of CSC due to mobilizing agents is relatively small (below 10%; Supplementary Section S7 and Figs. S12, S13). In addition, if mobilization agents are administered over multiple days, the malignant cell burden increases. The reason for this is that the CSC detaching from the niche are prone to divide and thus lead to an expansion of the malignant cell population.

The same observations apply to the setting of HSC gene therapy. In unpreconditioned hosts the homing of engineered cells improves if the transplantation is preceded by the administration of mobilizing agents; however, the benefit is negligible in preconditioned patients (Supplementary Section S7, Fig. S14).

## Discussion

The stem cell niche is closely intertwined with maintenance, repair, and malignancy of different tissues.^1,60^ Despite recent experimental achievements,^5,10,14^ we still lack a mechanistic understanding of its role in health and disease. We propose a mathematical model to provide insights into the mechanisms by which the stem cell niche governs population dynamics of healthy and malignant cells. In the model, HSC detach and re-attach to a bone marrow niche of finite capacity. In agreement with experimental findings, HSC are quiescent as long as they are attached to the niche.^45,61,62^ Detachment from the niche triggers activation of HSC which is followed by differentiation or division.^11,45-47^ To maintain the stem cell fate, HSC must reattach to the niche after division, otherwise they irreversibly differentiate. The model was parameterized based on mouse experiments from literature. In the model the homeostatic state corresponds to a dynamic equilibrium of stem cell attachment, stem cell detachment, stem cell gain due to proliferation, and stem cell loss due to differentiation.

Disease-related or experimentally induced alterations of HSC properties may lead to a contrary effect on the short compared with the long time scale: A persistent increase of the HSC attachment rate triggers a transient decline of circulating HSC followed by a durable increase. The initial decline can be explained by an increased niche attachment of circulating cells. Since niche attachment reduces the probability of HSC death or differentiation, the total number of HSC increases over time. In case of unchanged detachment rate, the number of HSC leaving the niche per unit of time increases if the number of niche-bound cells increases. This drives the system to a new equilibrium with increased niche-bound and circulating HSC. Analogously, in response to a persistent increase of the detachment rate, the homeostatic counts of unbound/circulating (actively cycling^45^) HSC increases, whereas the number of niche-bound cells remains unchanged. These observations suggest that we cannot infer the number of niche-bound HSC based on circulating HSC counts. Similar findings hold for progenitor and precursor cells. Consequently, peripheral blood or bulk marrow samples might provide only insufficient information about the stem cell compartment.

In the presence of multiple clones, we observe that a high number of progenitors or mature cells can arise from a small number of stem cells and vice versa, depending on the respective rates of stem cell proliferation, differentiation detachment and attachment. Consequently, the variant allele frequencies among progenitor, precursor, or mature cells may not correlate with the frequencies of mutated HSC. This conclusion agrees with recent results.^7,15^ Of note, in MPN patients, the abundance of the *JAK2V617F* or *CALR* mutations in the peripheral blood reflects the state of the marrow.^57,63^ Nevertheless this may not hold for other diseases and may also depend on the specific mutational background.

Our observation that small stem cell differentiation and high attachment rates imply a growth advantage agrees with previous results.^17,33,64^ In addition to this, our model suggests that the fitness of a clone is independent of its detachment rate.

The analysis of our model leads to the hypothesis that for a monoclonal population the homeostatic fraction of vacant niches can be used to quantify the stem cell fitness. If this is correct, the fitness of a given clone can be assessed in a non-competitive setting by quantifying the percentage of empty niche spaces, similar as it was done in the work of Bhattacharya et al.^65^ According to the predictions of our model, the clone with a lower amount of empty niche spaces in equilibrium will outgrow the clone with a higher number of empty niche spaces in equilibrium if both clones compete inside the same host, eg, after a transplantation or after CRISPR-based gene editing. This is a new and potentially testable hypothesis. Alternative potential scenarios to quantify the competitive advantage of a clone include xenotransplantation assays, humanized ossicles,^66^ or *in vitro* niches. Another testable hypothesis from our study is that mobilizing agents could support the eradication of malignant clones or, in the context of gene therapy, the engraftment of genetically engineered stem cells.

This work is based on several simplifications. For instance, the detachment rate is assumed to be constant in time. To simulate the effect of mobilizing agents, we increase the detachment rate as long as the drug is available. This is a simplification, since HSC mobilization may depend on endogenous signals which change over time.^67^ Another simplification is the consideration of the stem cell niche as a homogeneous entity neglecting the existence of perivascular and endosteal niches. Furthermore, we do not account for the turnover of the cells which form the non-hematopoietic part of the stem cell niche. If experimental data on the respective processes become available the model can be extended accordingly.

The experimental quantification of stem cell numbers and properties crucially depends on the approaches used and the obtained results can vary considerably.^54,65^ Therefore, the parameterization of our model is rather a proof of principle than a definitive quantification. So far, our model has been parameterized using murine data. According to our current knowledge, the qualitative dynamics could be similar in humans. Previous works combining mathematical models and human data support the concept that wild-type and mutated cells compete for space in a joined niche.^6,7^ Models are important tools to understand and optimize clinical procedures.^7,20,33,38,60^ However, our model must be carefully validated with clinical data before it can be used for medical purposes, such as treatment simulation.

## Conclusion

We have developed a mechanistic mathematical model to investigate how processes in the HSC niche impact clonal competition and dynamics of myeloid malignancies. The model suggests that rates of HSC proliferation, self-renewal, differentiation, and attachment to the niche are key determinants of a clone’s fitness. Based on model analysis and simulations, we suggest that the frequency of unoccupied niche spaces under homeostatic conditions might be a measure for the fitness of the HSC population. If true, this approach could help to experimentally determine the fitness of a monoclonal HSC population in a culture or xeno-transplantation model. Simulations of model dynamics imply that the abundance of malignant cells in the peripheral blood or in the precursor and progenitor compartments may substantially differ from the abundance of malignant clones in the stem cell niche. Depending on malignant cell parameters the frequency of malignant cells in the stem cell niche may grow faster or slower compared to downstream cell compartments. This finding may have implications for the interpretation not only of peripheral blood cell counts (as already known by clinicians) but also of unsorted bone marrow samples, which are part of the state-of-the-art diagnostics of many diseases. Since processes in the HSC niche can be modified by mobilizing agents, their quantitative understanding may have implications for future treatment approaches.

## Supplementary Material

Supplementary material is available at *Stem Cells* online.

sxac079_suppl_Supplementary_Materials

## Data Availability

The data underlying this article will be shared on reasonable request to the corresponding author.
