# Supplementary material for: HSC Niche Dynamics in Regeneration, Pre-malignancy, and Cancer: Insights From Mathematical Modeling
Source: Stem Cells. 2022 Nov 13;41(3):260–70. doi: 10.1093/stmcls/sxac079 (PMC10020982; doi:10.1093/stmcls/sxac079)
Supplement: sxac079_suppl_Supplementary_Materials [file sxac079_suppl_supplementary_materials.pdf]

Supporting Information

This Supporting Information contains the detailed model derivation, mathematical model analysis, model parameterization and Supplemental Figures.

Contents

S1 Model development . . . 1
S1.1 Model of HSC - niche interactions . . . 1
S1.2 Model for competition of multiple stem cell clones . . . 2
S1.3 Equivalence of models with and without asymmetric HSC divisions . . . 4
S1.4 Remarks on model design . . . 5
S2 Parameterization . . . 8
S3 Basic model properties . . . 12
S3.1 Equilibria . . . 12
S3.2 HSC dynamics in absence of niche cells . . . 12
S3.3 Response to perturbations of homeostatic cell counts . . . 12
S3.4 Impact of model parameters on equilibrium HSC counts and progenitor production . . . 12
S4 Model Analysis . . . 14
S4.1 Steady states . . . 14
S4.2 Parameter dependence of steady state cell counts . . . 17
S4.3 Parameter dependence of steady state progenitor production . . . 18
S4.4 Stability analysis . . . 18
S5 Peripheral blood malignant cell burden can change faster or slower compared to the malignant cell burden in the stem cell niche . . . 22
S6 Multiple stem cell doses increase chimerism in the unpreconditioned host but not in the preconditioned host . . . 22
S7 Mobilizing agents could decrease the residual disease in the unpreconditioned host but lead to an increase in the preconditioned host. . . . 25

S1 Model development

S1.1 Model of HSC - niche interactions

In this section we develop a mathematical model describing the interaction of HSC with the bone marrow niche. The model is based on the following biological processes.

1. HSC require interaction with the bone marrow microenvironment to maintain their stemness [20, 41, 49]. HSC attached to the niche are in a quiescent state, i.e., they do not divide [21]. This assumption is supported by the finding that niche-specific molecules such as osteopontin or CXCR4 inhibit entry into the cell cycle [28, 29, 19, 39, 21, 9].

2. HSC detach from the niche at a rate u_H. Upon detachment HSC become activated. This asumption is in line with experiments showing that an interruption of CXCR4 or osteopontin related queues leads to cell cycle entry [28, 29, 39]. This transition can be written as

$$\mathcal{N}_{\mathcal{H}} \overset{u_H}{\to} \mathcal{A}_{\mathcal{H}} + \mathcal{N}_{\mathcal{E}}, \tag{S1}$$

where $\mathcal{N}_{\mathcal{H}}$ denotes a niche space that is occupied by an HSC, $\mathcal{A}_{\mathcal{H}}$ denotes an activated HSC and $\mathcal{N}_{\mathcal{E}}$ a vacant niche space. For simplicity, we assume that HSC activation occurs immediately after detachment.

3. Activated HSC can divide and differentiate into progenitors at a rate d_{A_H}. This transition is written as

$$\mathcal{A}_{\mathcal{H}} \overset{d_{A_H}}{\to} 2\mathcal{D}_{\mathcal{H}}, \tag{S2}$$

corresponding to the loss of a stem cell, $\mathcal{D}_{\mathcal{H}}$ denotes a progenitor.

4. Activated HSC can divide at a rate r_H. We assume that the offspring originating from division are inactive HSC, i.e., they have to reattach to the niche before they can divide again. Inactive HSC either differentiate at a rate d_{I_H} or reattach to the niche at a rate b_H. We assume that inactive cells cannot be activated outside the niche. This implies that HSC differentiate in absence of the stem cell niche, as it is observed in cell culture experiments [20, 41, 49]. For the sake of simplicity we assume that activated

HSC become inactive after performing one division. It is straightforward to extend the model to allow an arbitrary finite number of divisions before becoming inactive. On the population level it makes no difference whether we allow asymmetric stem cell divisions or not. In section S1.3 we formally show that the model introduced here based on symmetric HSC divisions is equivalent to a model including both symmetric and asymmetric HSC divisions. The described processes can be written as

$$\mathcal{A}_{\mathcal{H}} \overset{r_H}{\to} 2\,\mathcal{I}_{\mathcal{H}} \tag{S3}$$

$$\mathcal{I}_{\mathcal{H}} \overset{d_{I_H}}{\to} \mathcal{D}_{\mathcal{H}} \tag{S4}$$

$$\mathcal{I}_{\mathcal{H}} + \mathcal{N}_{\mathcal{E}} \overset{b_H}{\to} \mathcal{N}_{\mathcal{H}}, \tag{S5}$$

where $\mathcal{I}_{\mathcal{H}}$ denotes an inactive HSC.

5. The progenitors, $\mathcal{D}_{\mathcal{H}}$, are committed to differentiation and cannot rebind to the niche. Instead of explicitly modeling progenitor dynamics, we assume that the mature cell output is correlated with progenitor production. This is reasonable since progenitors can only perform a limited number of divisions and thus each progenitor gives rise to an approximately constant number of mature cells.

6. For the sake of simplicity we assume that the bone marrow niche has a constant finite capacity, denoted by K [48].

We neglect re-attachment of stem cells to the niche without division. Simulations and mathematical analysis suggest that this simplification does not impact model dynamics. Similarly, allowing more than one division of activated cells before inactivation has no impact dynamics in the stem cell niche [31]. The biological processes underlying the model are summarized in Figure 1 of the main text and in Figure S1. We assume that the cells are well-mixed. This view is supported by magnetic resonance imaging of acute myeloid leukemia patients who mostly show a non-focal diffuse marrow infiltration and by post-mortem biopsy studies of chronic myeloproliferative diseases [40, 12]. This fits well to the concept that malignant cells spread through the blood stream to various bone marrow sites. Due to the high number of cells in the hematopoietic system [1, 44] we describe population dynamics using ordinary differential equations (ODEs). We denote by A_H the abundance (measured e.g., as cells per kg of body weight) of activated HSC, by I_H that of inactive HSC, by N_H that of occupied niche spaces and by N_E that of empty niche spaces. The influx to the progenitor compartment per unit of time is denoted i_{D_H}.

Based on the processes described above we obtain the following system of ODEs

$$\frac{dN_H}{dt} = b_H(K - N_H)I_H - u_H N_H \tag{S6a}$$

$$\frac{dI_H}{dt} = -b_H(K - N_H)I_H + 2r_H A_H - d_{I_H} I_H \tag{S6b}$$

$$\frac{dA_H}{dt} = u_H N_H - r_H A_H - d_{A_H} A_H \tag{S6c}$$

$$N_E = K - N_H \tag{S6d}$$

$$i_{D_H} = 2d_{A_H} A_H + d_{I_H} I_H \tag{S6e}$$

supplemented by the initial conditions $N_H(0) \in [0, K]$, $I_H(0) \geq 0$ and $A_H(0) \geq 0$.

For brevity, we refer to the sum of I_H and A_H as 'free HSC'.

S1.2 Model for competition of multiple stem cell clones

In this section we extend the model to account for clonal competition.

Clinically relevant conditions such as clonal hematopoiesis, AML or MPN are related to the presence of multiple stem cell clones [44, 18, 6, 32, 26, 36, 38]. In this section we extend the model to take into account such scenarios. For the sake of simplicity we consider two stem cell populations with different properties. In case of clonal hematopoiesis these two stem cell populations correspond to two HSC clones with slightly different properties. In case of malignancy the two clones correspond to healthy (HSC) and malignant or leukemic stem cells (LSC). In a setting after gene therapy the cell populations correspond to host-derived wild-type and transplant-derived transduced cells. Extension of the model to more than two clones is straightforward but will

not be considered in this work. In the model both stem cell types reside in the same niche and compete for niche spaces, similar to the work of Wang et al. [44], Stiehl et al. [38] and Ashcroft et al. [3].

The model is visualized in Figure S1. For the sake of convenience we denote one cell type by H and the other by L.

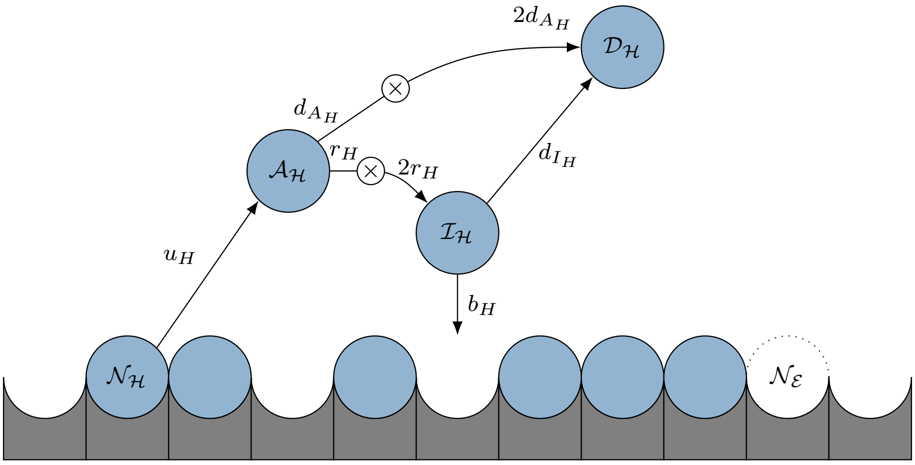

(a) **Homogeneous stem cell population.** HSC detachment from the niche occurs at rate u_H, division of activated stem cells followed by differentiation at rate d_{A_H}, differentiation of inactive stem cells at rate d_{I_H}, division of active stem cells at rate r_H and attachment of inactive stem cells to the niche at rate b_H. Counts of niche-bound cells are denoted N_H, counts of inactive cells as I_H and counts of activated stem cells as A_H.

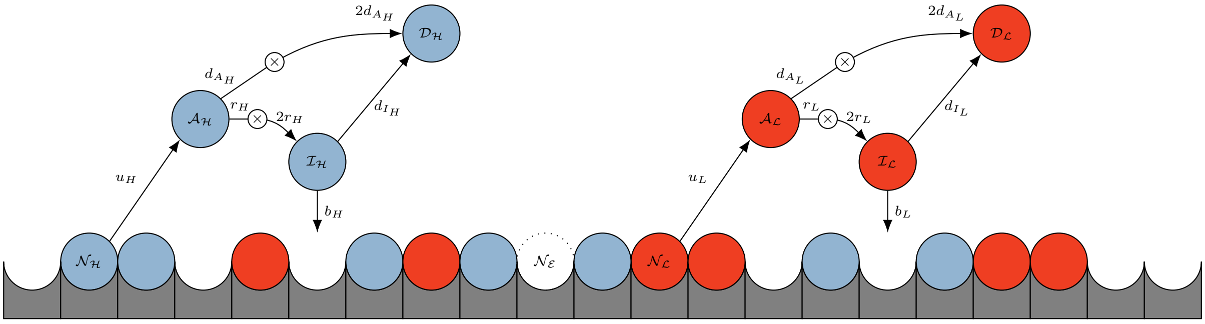

(b) **Competition of two stem cell clones.** Subscript H denotes the first (e.g., healthy) clone, while L denotes the second (e.g., leukemic) clone.

Figure S1: **Schematic representation of the model.** (a) Homogeneous stem cell population, corresponding to a healthy scenario. (b) Competition of two stem cell populations, corresponding to AML, MPN, clonal hematopoiesis or following transplantation of donor cells or genetically engineered cells. In panel (b), we use the abbreviations introduced in panel (a). Empty niches are denoted by N_E. The index H refers to healthy cells, the index L to donor-related, mutated or malignant cells.

In analogy to the healthy system described in section S1.1, both clones are subjected to the following biological processes.

1. Stem cells attached to the niche are quiescent and upon detachment they become activated. This assumption is motivated by the observation that malignant cells also enter cell cycle if they are mobilized from the niche or if niche-related signaling is disrupted [32, 34, 33, 46]. HSC and LSC detach from the niche at rates u_H and u_L respectively:

$$\mathcal{N_H} \xrightarrow{u_H} \mathcal{A_H} + \mathcal{N_E} \tag{S7}$$

$$\mathcal{N_L} \xrightarrow{u_L} \mathcal{A_L} + \mathcal{N_E}, \tag{S8}$$

   where $\mathcal{N_L}$ denotes a niche space that is occupied by a LSC and $\mathcal{A_L}$ denotes an activated LSC.

2. Activated stem cells divide and differentiate at rates d_{A_H} and d_{A_L}. This can be written as

$$\mathcal{A}_{\mathcal{H}} \overset{d_{A_H}}{\to} 2\mathcal{D}_{\mathcal{H}} \tag{S9}$$

$$\mathcal{A}_{\mathcal{L}} \overset{d_{A_L}}{\to} 2\mathcal{D}_{\mathcal{L}}, \tag{S10}$$

corresponding to the loss of stem cells. Here, $\mathcal{D}_{\mathcal{L}}$ denotes a LSC-derived progenitor.

3. Activated cells divide into two inactive cells at rates r_H and r_L respectively. Inactive cells can either differentiate (at rates d_{I_H} and d_{I_L}) or reattach to the niche (at rates b_H and b_L):

$$\mathcal{A}_{\mathcal{H}} \overset{r_H}{\to} 2\,\mathcal{I}_{\mathcal{H}} \tag{S11}$$

$$\mathcal{I}_{\mathcal{H}} \overset{d_{I_H}}{\to} \mathcal{D}_{\mathcal{H}} \tag{S12}$$

$$\mathcal{I}_{\mathcal{H}} + \mathcal{N}_{\mathcal{E}} \overset{b_H}{\to} \mathcal{N}_{\mathcal{H}} \tag{S13}$$

$$\mathcal{A}_{\mathcal{L}} \overset{r_L}{\to} 2\,\mathcal{I}_{\mathcal{L}} \tag{S14}$$

$$\mathcal{I}_{\mathcal{L}} \overset{d_{I_L}}{\to} \mathcal{D}_{\mathcal{L}} \tag{S15}$$

$$\mathcal{I}_{\mathcal{L}} + \mathcal{N}_{\mathcal{E}} \overset{b_L}{\to} \mathcal{N}_{\mathcal{L}}, \tag{S16}$$

where $\mathcal{I}_{\mathcal{L}}$ denotes an inactive LSC.

Denoting by A_L the abundance (measured e.g., as cells per kg of body weight) of activated LSC, by I_L that of inactive LSC, by D_L that of LSC-derived progenitors and by N_L that of niches occupied by LSC we obtain the following system of ODEs:

$$\frac{dN_H}{dt} = b_H(K - N_H - N_L)I_H - u_H N_H \tag{S17a}$$

$$\frac{dI_H}{dt} = -b_H(K - N_H - N_L)I_H + 2r_H A_H - d_{I_H} I_H \tag{S17b}$$

$$\frac{dA_H}{dt} = u_H N_H - r_H A_H - d_{A_H} A_H \tag{S17c}$$

$$\frac{dN_L}{dt} = b_L(K - N_H - N_L)I_L - u_L N_L \tag{S17d}$$

$$\frac{dI_L}{dt} = -b_L(K - N_H - N_L)I_L + 2r_L A_L - d_{I_L} I_L \tag{S17e}$$

$$\frac{dA_L}{dt} = u_L N_L - r_L A_L - d_{A_L} A_L \tag{S17f}$$

$$N_E = K - N_H - N_L, \tag{S17g}$$

$$i_{D_H} = 2d_{A_H} A_H + d_{I_H} I_H, \tag{S17h}$$

$$i_{D_L} = 2d_{A_L} A_L + d_{I_L} I_L \tag{S17i}$$

with the initial conditions $N_L(0) \geq 0$, $N_H(0) \geq 0$, $N_L(0) + N_H(0) \leq K$, $A_H(0) \geq 0$, $I_H(0) \geq 0$, $A_L(0) \geq 0$, $I_L(0) \geq 0$.

In the model both stem cell clones compete for the same niche spaces. Depending on the cell properties (proliferation rate, attachment rate, detachment rate etc.) one clone can out-compete the other clone from the niche.

S1.3 Equivalence of models with and without asymmetric HSC divisions

In this section we show that it has no impact on system dynamics whether HSC can divide asymmetrically or not.

In the model derivation we have assumed that HSC always divide symmetrically (point 4 in section S1.1). In the following we show that up to a reparametrization, this is equivalent to a model allowing symmetric and asymmetric HSC divisions.

In a scenario where asymmetric HSC divisions are possible, the processes

$$\mathcal{A}_{\mathcal{H}} \overset{r_H}{\to} 2\mathcal{I}_{\mathcal{H}} \tag{S18}$$

$$\mathcal{A}_{\mathcal{H}} \overset{d_{A_H}}{\to} 2\mathcal{D}_{\mathcal{H}} \tag{S19}$$

are replaced by

$$\mathcal{A}_{\mathcal{H}} \overset{S_r}{\to} 2\mathcal{I}_{\mathcal{H}} \tag{S20}$$

$$\mathcal{A}_{\mathcal{H}} \overset{a}{\to} \mathcal{I}_{\mathcal{H}} + \mathcal{D}_{\mathcal{H}} \tag{S21}$$

$$\mathcal{A}_{\mathcal{H}} \overset{S_d}{\to} 2\mathcal{D}_{\mathcal{H}} \tag{S22}$$

where S_r is the rate of symmetric self-renewing divisions, S_d the rate of symmetric divisions resulting in differentiated cells, and a is the rate of asymmetric divisions.

The full mathematical model with explicit asymmetric divisions is then

$$\frac{dN_H}{dt} = b_H(K - N_H)I_H - u_H N_H \tag{S23a}$$

$$\frac{dI_H}{dt} = -b_H(K - N_H)I_H + (2S_r + a)A_H - d_{I_H} I_H \tag{S23b}$$

$$\frac{dA_H}{dt} = u_H N_H - (S_r + a + S_d)A_H \tag{S23c}$$

$$N_E = K - N_H \tag{S23d}$$

$$i_{D_H} = (2S_d + a)A_H + d_{I_H} I_H \tag{S23e}$$

Defining $r_H = S_r + \frac{a}{2}$, we have $\frac{dI_H}{dt} = -b_H(K - N_H)I_H + 2r_H A_H - d_{I_H} I_H$ as in equations (S6). This implies that

$$\frac{dA_H}{dt} = u_H N_H - (S_d + 2r_H - S_r)A_H \tag{S24}$$

$$i_{D_H} = (2S_d + 2r_H - 2S_r)A_H + d_{I_H} I_H. \tag{S25}$$

Defining $d_{A_H} = S_d + r_H - S_r = S_d + \frac{a}{2}$ yields exactly the system of equations as (S6). This demonstrates the equivalence of the model with and the model without asymmetric divisions.

Later, we will see that $r_H > d_{A_H}$ is a necessary condition for the existence of a positive homeostatic equilibrium (see equations (S30) and (S26)). We note that the inequality $r_H > d_{A_H}$ is equivalent to $S_r > S_d$, since $d_{A_H} = S_d + r_H - S_r$. Therefore, this condition can be interpreted such that the rate of symmetric self-renewing divisions, S_r, must exceed the rate of symmetric differentiating divisions, S_d, regardless of the absence or presence of asymmetric divisions.

S1.4 Remarks on model design

In this section we motivate why our model distinguishes between activated and inactive stem cells.

We aim to derive a model that is in line with the following experimental and clinical observations:

(i) Stem cells in the niche are quiescent i.e., they do not divide

(ii) Stem cells outside the niche can divide and/or differentiate

(iii) The system has a homeostatic equilibrium, i.e., an equilibrium where cell counts are positive

(iv) The system can recapitulate bone marrow transplantation, i.e., if a small number of HSC is added to empty niches the HSCs expand.

(v) In absence of a stem cell niche the number of stem cells declines over time.

In the following we consider different candidate models which are summarized in Figure S2.

Candidate Model 1
Let us consider a niche of fixed capacity K. We distinguish between non-dividing stem cells in the niche (N) and active stem cells outside the niche (A). Stem cells in the niche die/differentiate at rate μ and detach from the niche at a rate u. Stem cells outside the niche are activated to divide at rate r and then divide into two

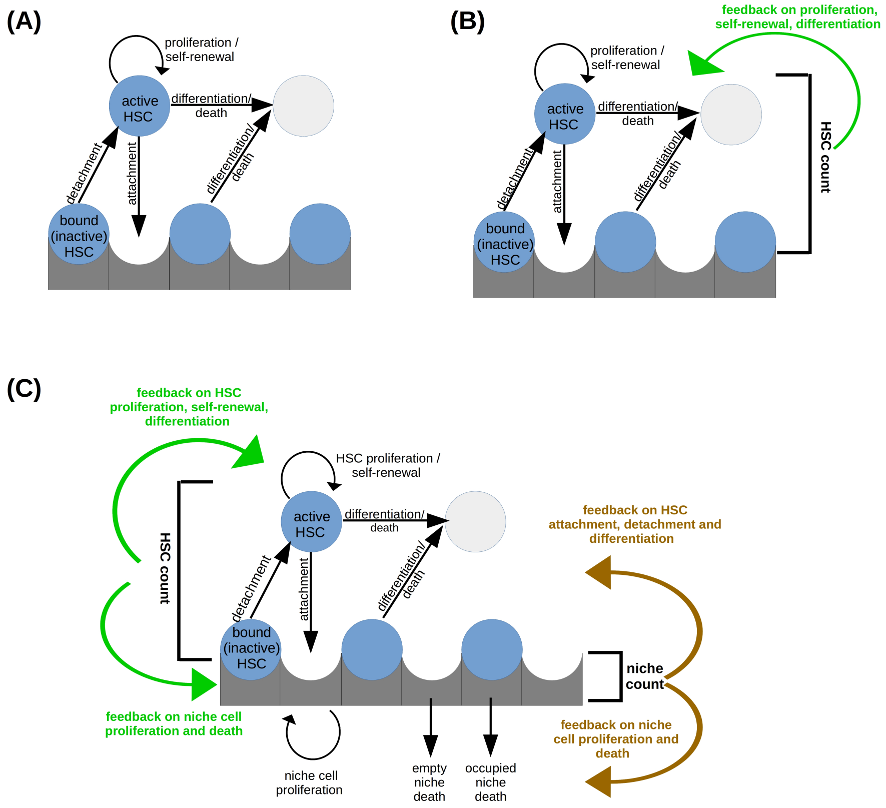

Figure S2: Different candidate models which were ruled out because they contradict clinical and experimental observations. (A) Model of the stem cell niche, where cells inside the niche are quiescence and cells outside the niche are activated. This model corresponds to the model from section S1.1, however without the distinction between activated and inactive HSC. (B) Model from (A) with feedback regulation. Proliferation, differentiation and death of stem cells are regulated by the stem cell number (e.g., total stem cell count, number of active stem cells or number of stem cells in the niche). (C) Model of the stem cell niche. The stem cell niche can grow and shrink dependent on the number of stem and niche cells. Stem cell attachment, detachment, proliferation and loss can depend on feedback signals.

offspring. Cells outside the niche are removed at rate d due to death or differentiation. The attachment of cells is proportional to the product of free niche spaces $K-N$ and cells outside the niche A. The proportionality factor is referred to as b. The model is illustrated in Figure S2 (A). These assumptions lead to the following equations.

$$\frac{dA}{dt} = uN + (2r-1)A - dA - b(K-N)A$$
$$\frac{dN}{dt} = b(K-N)A - uN - \mu N,$$

with $u,\ r,\ b,\ K, d,\ > 0,\ \mu \geq 0$.

We linearize around the trivial equilibrium $A=0$, $N=0$ and obtain the following characteristic polynomial

$$\chi(X) = X^2 + \big(u + \mu + bK + d - (2r-1)\big)X + \Big(\big(d-(2r-1)\big)(u+\mu) + bK\mu\Big).$$

If $\big(d-(2r-1)\big) > 0$ the characteristic polynomial has two roots with negative real parts, i.e., the trivial steady state is not unstable. This contradicts (iv). For simplicity, we neglect the case $\big(d-(2r-1)\big) = 0$, which is improbable to be biologically realized since it is prone to be disturbed by noise. Therefore, we consider $\big(d-(2r-1)\big) < 0$.
In absence of the niche, the dynamics of stem cells is governed by

$$\frac{dA}{dt} = \big((2r-1) - d\big)A,$$

which implies exponential growth of HSC. This contradicts to (v). Therefore, this model cannot satisfy (i)-(v).

Candidate Model 2:
We consider a more general form of candidate model 1. We assume that proliferation and differentiation of cells outside the niche and clearance of stem cells in the niche are regulated by a feedback signal that depends on a weighted sum of A and N. The weights are denoted by $\alpha, \beta \geq 0$. The model is illustrated in Figure S2 (B). This results in the following equations.

$$\frac{dA}{dt} = f(\alpha A + \beta N)A + uN - b(K-N)A$$
$$\frac{dN}{dt} = b(K-N)A - uN - \mu(\alpha A + \beta N)N$$

The term $f(\alpha A + \beta N)A$ denotes the difference of the influx of unbound stem cells due to proliferation and the outflux due to differentiation and death. The term $\mu(\alpha A + \beta N) \geq 0$ describes the loss of niche-bound stem cells due to differentiation and death. Both are assumed to be continuously differentiable. For $f(\alpha A + \beta N) = (2r-1) - d$, $\mu(\alpha A + \beta N) = \mu$ we obtain candidate model 1. This model covers regulations of proliferation and differentiation by Hill-type functions such as in [24, 11]. For $\alpha = \beta = 1$ the feedback depends on the total stem cell count, for $\alpha = 0,\ \beta = 1$ the feedback depends on the number of stem cells in the niche and for $\alpha = 1, \beta = 0$ it depends on stem cells outside the niche.

We linearize around the trivial equilibrium and obtain

$$\chi(X) = X^2 + \big(u + \mu(0) + bK - f(0)\big)X + \Big(-f(0)(u + \mu(0)) + bK\mu(0)\Big).$$

If $f(0) < 0$, the characteristic polynomial has two roots with negative real parts and the trivial equilibrium is not unstable. This contradicts to (iv). For simplicity, we neglect the case $f(0) = 0$, which is improbable to be biologically realized since it is prone to be disturbed by noise. Therefore, assume $f(0) > 0$. Then we obtain the following dynamics in absence of the niche ($N = K = 0$):

$$\frac{d}{dt}A = f(\alpha A)A$$

The linearization of the right hand-side around $A = 0$ is $f(0)$, which is positive. This implies that the stem cell count cannot converge to zero in absence of the niche, which violates (v). Therefore, this approach cannot satisfy (i)-(v).

Candidate Model 3:
We consider a scenario where the number of niches is not constant but depends on the stem cell counts. Denote by N_E the number of empty niches, by N the number of stem cells residing in niches, i.e., the total number of niches is $N_E + N$. Denote by A the number of stem cells detached from the niche.

Denote the production of empty niches by $f(N_E, N, A) \geq 0$. It might be positive even if $N_E = 0$, since niche cells may be produced by non-niche cells. Denote the decay of empty niches by $\mu(N_E, N, A) \geq 0$. The attachment of stem cells to the niche is proportional to the product of free niche spaces N_E and cells outside the niche A. The proportionality factor is referred to as b. The detachment rate is denoted by $u(N_E, N, A) \geq 0$. Death or differentiation of HSC in the niche occurs at rate $g(N_E, N, A) \geq 0$. Death of niche cells which are attached to HSCs occurs at rate $h(N_E, N, A) \geq 0$. Death or differentiation of stem cells outside the niche occurs at rate $d(N_E, N, A) > 0$. We assume that the stem cell proliferation rate r may be regulated by a feedback depending on the weighted sum of stem cells and set $r \equiv r(\alpha A + \beta N)$ for $\alpha, \beta \geq 0$. The model is illustrated in Figure S2 (C). This results in the following system of equations. All coefficients are assumed to be continuously differentiable functions.

$$\frac{d}{dt}N_E = f(N_E, N, A) - b(N_E, N, A)AN_E + u(N_E, N, A)N - \mu(N_E, N, A)N_E + g(N_E, N, A)N$$
$$\frac{d}{dt}A = r(\alpha A + \beta N)A - b(N_E, N, A)AN_E + u(N_E, N, A)N + h(N_E, N, A)N - d(N_E, N, A)A$$
$$\frac{d}{dt}N = b(N_E, N, A)AN_E - u(N_E, N, A)N - h(N_E, N, A)N - g(N_E, N, A)N$$

For the total number of stem cells it holds

$$\frac{d}{dt}(A + N) = r(\alpha A + \beta N)A - d(N_E, N, A)A - g(N_E, N, A)N \leq r(\alpha A + \beta N)A.$$

If it holds $r(0) < 0$, a small amount of transplanted cells will not expand. We therefore assume $r(0) > 0$. For simplicity, we neglect the case $r(0) = 0$, which is improbable to be biologically realized since it is prone to be disturbed by noise. In absence of the niche ($N = N_E = 0$), stem cell dynamics are governed by

$$\frac{d}{dt}A = r(\alpha A)A,$$

which is unstable in $A = 0$, which means that stem cells can survive without the niche. This is a contradiction.

Conclusion
Since these straightforward models violate biological assumptions, we introduce the inactive stem cell state.

The reason why we introduce the inactive state is the observation that HSCs cannot expand or be maintained in absence of the stem cell niche [20, 41, 49]. In a model without the inactive state, where stem cells are either attached to the niche (and quiescent) or detached from the niche (and active) we would observe a self-maintaining population of active stem cells, even if the niche is removed. The inactive state implies that in absence of the stem cell niche all stem cells eventually differentiate. The inactive state in the model corresponds to early differentiation events (which are still reversible if the cell rebinds to the niche). This state may be linked to changes of gene expression occurring if stem cells expand outside the niche, as they have been described in [14] or as they occur in the case of niche impairment [43].

S2 Parameterization

In this section we fit the model to experimental data and set the model parameters for further simulations.

The quantification of stem cell numbers is challenging and different approaches have been developed. The estimates of stem cell number in an individual mouse are approximately $5000 - 15000$, depending on age, sex, strain and methodology [25, 10]. The percentage of empty niches can be quantified using unconditioned transplants, as described in [5], which suggests that in equilibrium $0.1 - 1\%$ of niches are empty. The same study suggests that approximately $1-5\%$ of HSC enter blood stream each day. Parabiosis experiments in mice suggest that $5 - 10\%$ of HSC travel in bloodstream within 7 weeks [47]. A quantitative study of murine steady state hematopoiesis is given in a work by Busch et al. [8]. This work estimates a lower bound of 17,000 HSC per mouse (0.006% of bone marrow nucleated cells), 30% of which are active.

Quantifications in the human system are even more difficult. One possibility is to use CD34+CD38-ALDH+ cells [44]. This leads to approximately 10^7 HSC per kg of body weight. The number of human HSCs actively contributing to blood cell formation at a given time is estimated by 50,000–200,000 based on somatic mutations [23]. HSC mobilization studies in humans show that the number of circulating HSCs can increase more than 50 fold [17], implying that less than 2% of HSC are circulating under equilibrium conditions. After bone-marrow transplantation the average time HSC spend in the blood stream is approximately one day [15].

In the following we calibrate our proposed model to the murine system. The number of niches is set to $K = 15000$, based on estimations in the work of Catlin et al. [10]. Model dynamics remain qualitatively unchanged if different values for K are chosen.

Stem cell niche dynamics can be experimentally studied based on transplantation of stem cells in unconditioned hosts [4]. The work from [4] provides evidence that consecutive transplantation of small HSC doses (12700/7 cells on 7 consecutive days) leads to an increased chimerism compared to the transplantation of the total cell dose (12700 cells) at a single time point. This observation suggests that transplanted stem cells home to niche spaces that are unoccupied under steady state conditions. To re-equilibrate the system releases random (i.e., donor and host-derived) stem cells from the niche. In case of sequential transplantations this process is reiterated and donor-derived stem cells home to the niche at the expense of the randomly released host-derived cells. As a proof of concept, we demonstrate that our model can reproduce these experimental observations.

For this purpose we fit the model to the data of [4], using simulated annealing. The experimental data was extracted from Figure 6 of Bhattacharya et al. [4] based on visual inspection. Simulated annealing was performed using the MATLAB implementation by Vandekerckhove [42] with the following settings: Initial temperature: 10^3, Stop temperature: 10^{-3}, Max tries: 30. We assume that host and transplant cells have identical parameters, i.e. we set $r_H = r_L$, $d_{A_H} = d_{A_L}$, $u_H = u_L$, $b_H = b_L$ and $d_{I_H} = d_{I_L}$. Cell populations with index H are considered as host cells, cell populations with index L as cells of donor origin. At the time point of transplantation the host cell counts A_H, I_H, N_H are in equilibrium, and a number of free cells A_L and I_L is added, in accordance with the number of cells added in the work of Bhattacharya et al. [4]. As in the work of Stiehl et al. [37], we do not consider all transplanted cells to be stem cells, but reduce the number of cells added by one order of magnitude. Simulations indicate that if the transplanted cell count was much further reduced, the final chimerism in the model was smaller than in the experiments from Bhattacharya et al. [4], regardless of model parametrization. The transplanted stem cells added are assumed to be an equal mixture of activated and inactive cells, i.e., $A_L(0) = I_L(0)$¸where $t = 0$ is the time of transplantation. This assumption is made for the sake of simplicity since the impacts of stem cell harvesting and stem cell processing on HSC properties during transplantation are not well understood [45]. Based on our numerical analysis this assumption has no relevant impact on the obtained results. For comparison with experimental data we calculate the chimerism at time t as $\frac{N_L(t)}{N_H(t)+N_L(t)} \cdot 100\%$.

The parameters obtained from the simulated annealing are given in Table S1. The model can fit the experimentally observed short-term (one week) and long term (16 weeks) dynamics, however with different parameter sets. The results are shown in Figures S3 and S4. Qualitative model dynamics are similar for both parameter sets. All depicted simulations are based on the parameter set that matches long-term dynamics. Using the other parameter set leads to the same qualitative results. Our investigations did not find any parameter-set fitting both data-sets simultaneously. However, both parameter sets from the fitting match the data qualitatively, i.e., for both parameter sets multiple transplant doses imply an increased chimerism compared to the single dose scenario and the steady state value for the single dose scenario is identical for both parameter sets. A reason for the differences in the fitted parameters might be that in the model HSCs immediately home to the bone marrow after transplantation. Mechanisms related to homing that occur on the time-scale of a single day are not included in the model and could potentially explain the discrepancy. In addition, biological explanations for increasing uptake of subsequent transplants over time could also play a role, but are not considered in the model, since no short-term data of the multi-dose transplantation is available.

The number of HSC contained in $CD34^+$ cell grafts and the fraction that homes to the bone marrow cannot be exactly quantified. For simulations of clinical scenarios we assume, in agreement with clinical data, that a standard human graft contains $3 \cdot 10^3$ HSC per kg of body weight and that the remaining transplanted $CD34^+$ cells are progenitors [37]. We assume that HSC are equally distributed between the activated and the inactive state. Numerical simulations support that the qualitative results are robust with respect to changes in the number of transplanted activated and inactive HSC.

In formula (S43) we compute the steady state production of new progenitors, which is equal to the steady state division rate of stem cells under equilibrium conditions (since in equilibrium in average one dividing stem

cell gives rise to one stem cell and one progenitor). It turns out that the progenitor production and thus the stem cell division rate is equal to u_H, which in our estimates is, depending on the data used for the fit, between 1:25 and 1:250 per day. The stem cell birth/differentiation rate estimated by Busch et al [8] is 1:110 per day and is between our fits.

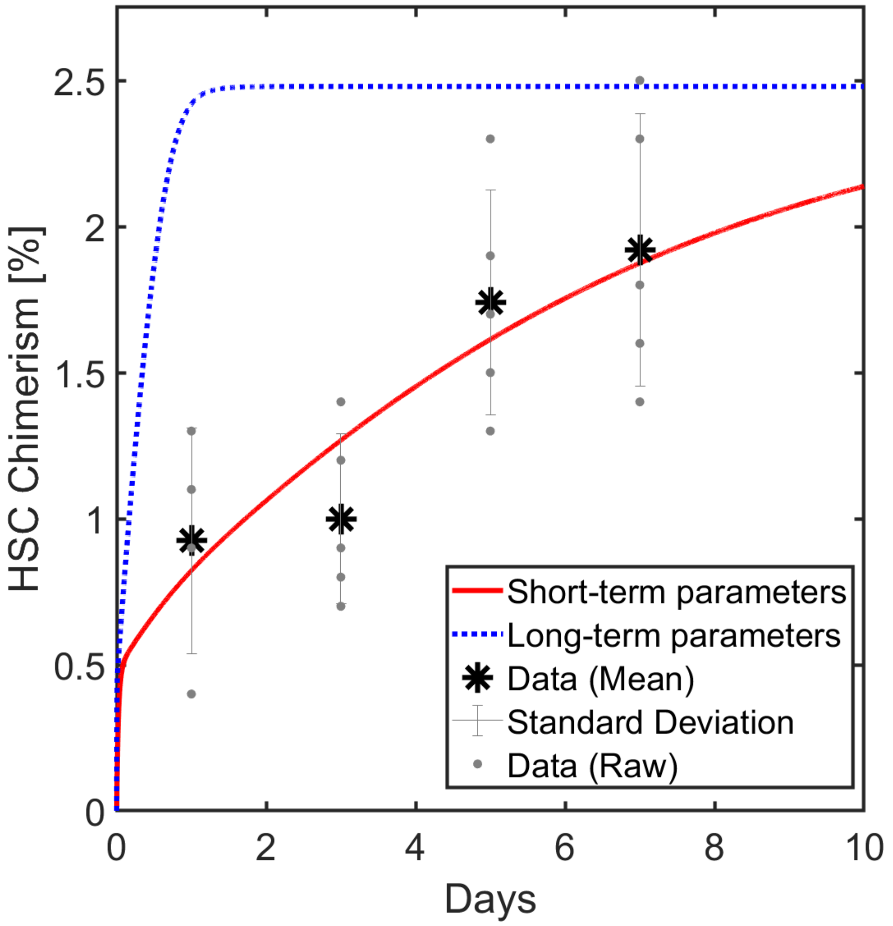

Figure S3: Simulated HSC chimerism (best fit) after single dose transplant in an unconditioned host. Black points depict the short-term data from Bhattacharya et al. [4]. The red line shows the fit of the model to the short-term data ($R^2 = 0.86$). Parameter values are provided in Table S1. The dotted line (long-term parameters) is shown for the purpose of comparison, it results from the fit of the model to the chimerism after 112 days, which is depicted in Fig. S4.

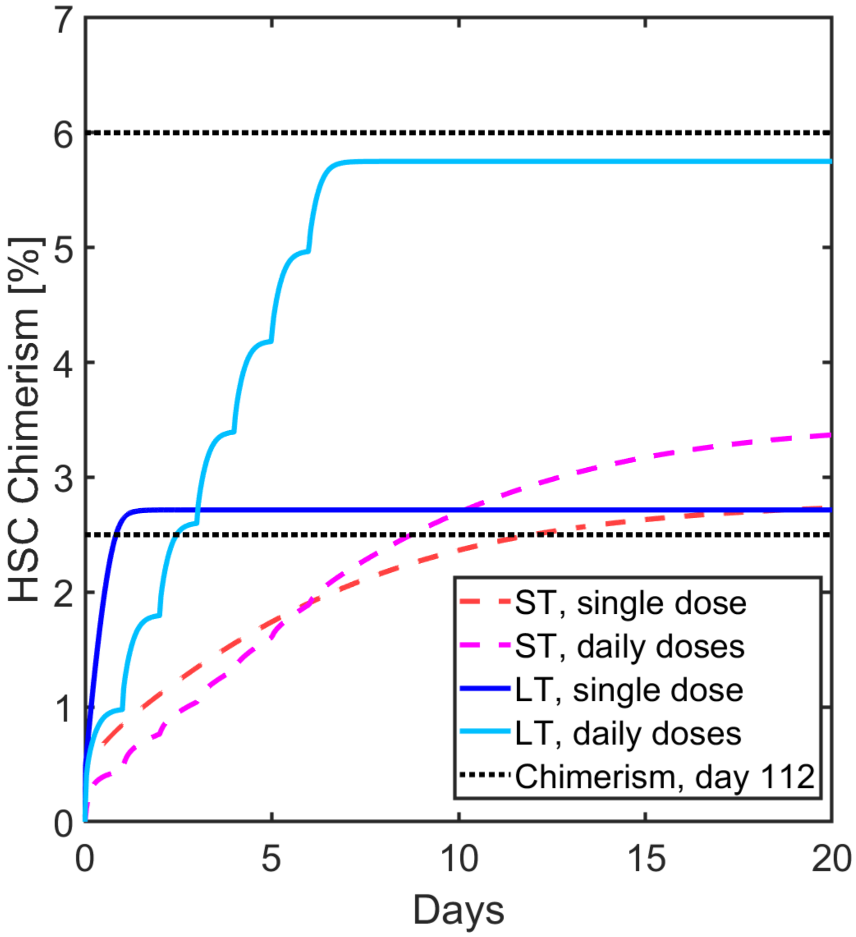

Figure S4: Simulated HSC chimerism (best fit) after 7 small transplants on consecutive days compared to a single dose transplant. Hosts are unconditioned. Black dotted lines correspond to the chimerism after 16 weeks (112 days) as measured in Bhattacharya et al. [4]. Parameter values are provided in Table S1. "LT" represents the fit of the model to the chimerism data at day 112, which results in the "long-term parameter set". "ST" is depicted for comparison and refers to model dynamics using the "short-term parameter set" obtained from fitting the model to the data depicted in Fig. S3.

Table S1: Best fit of parameter values found using simulated annealing. We assume that host cells and transplanted cells have the same parameters. Therefore u denotes both u_H and u_L in the simulation, and similarly for the other parameters.

Short-term				Long-term			
K	15000 cells	u	0.004 day^{-1}	K	15000 cells	u	0.04 day^{-1}
r	0.14 day^{-1}	b_I	0.09 day^{-1}	r	2.32 day^{-1}	b_I	0.96 day^{-1}
d_A	0.07 day^{-1}	d_I	2.39 day^{-1}	d_A	2.06 day^{-1}	d_I	3.77 day^{-1}

S3 Basic model properties

S3.1 Equilibria

In this section we summarize the equilibrium states of the model.

The model possesses the following four equilibrium states. The formal derivation is presented in section S4.1.

- **Stem cell free equilibrium:** $N_H = N_L = I_H = A_H = I_L = A_L = 0,\ N_E = K.$

  This equilibrium corresponds to the absence of stem cells and will lead to the death of the organism. This equilibrium also corresponds to the state after conditioning for bone marrow transplantation before the stem and progenitor cells are infused or to a situation with graft failure. This equilibrium exists for all parameter values.

- **Homeostasis:** $N_E > 0,\ N_H > 0,\ I_H > 0,\ A_H > 0,\ N_L = I_L = A_L = 0.$

  This equilibrium corresponds to the homeostatic state of a healthy organism. All cells are wild-type cells. As shown in section S4.1, this equilibrium only exists if activated stem cells divide more often than they differentiate and if generation of inactive stem cells outweighs their differentiation.

- **Out-competition of wild-type cells:** $N_E > 0,\ N_L > 0,\ I_L > 0,\ A_L > 0,\ N_H = I_H = A_H = 0.$

  This equilibrium corresponds to a state where all wild-type cells have been out-competed. In case of stem cell transplantation this means that all HSC are derived from the graft. In case of malignancy, this state corresponds to the out-competition of HSC by leukemic cells which leads to the death of the organism. In case of clonal hematopoiesis of indeterminate potential (CHIP), this corresponds to monoclonal hematopoiesis. This equilibrium exists only if activated mutated (or respectively transplanted) stem cells divide more often than they differentiate and if generation of inactive stem cells outweighs their differentiation.

- **Coexistence in equilibrium:** In this equilibrium all population counts are positive. This corresponds to coexistence of wild-type and other cells. This type of equilibrium exists only for very specific parameter choices which is biologically unlikely to be realized.

Detailed calculations are provided in supplementary section S4.1.

S3.2 HSC dynamics in absence of niche cells

In the absence of niche cells it holds $K = 0$ implying $N_H(0) = N_E(0) = 0$. Under this assumptions, as shown in Figure S5, A_H and I_H converge to zero. This is in line with experimental observations from cell culture experiments [20, 41, 49].

S3.3 Response to perturbations of homeostatic cell counts

After perturbations, e.g., by blood loss, immune reaction, radiation, chemotherapy or bone marrow transplantation the hematopoietic system comes back to its equilibrium state. This property is captured by our model. When slightly perturbing homeostatic cell counts, the system returns to the homeostatic state. In supplementary section S4.4 we prove this property rigorously. An exemplary simulation is provided in figure S6.

S3.4 Impact of model parameters on equilibrium HSC counts and progenitor production

In this section we study how the cell counts of the healthy equilibrium and the production rate of progenitors depend on model parameters.

Changes of parameter values affect homeostatic HSC counts. The parameter dependence of the steady state cell counts is mathematically studied in section S4.2 of this supplement and summarized in Table 1 of the main text. Figure S7 illustrates how sensitively homeostatic cell counts depend on perturbations of parameter values. The figure shows the impact of a 5% perturbation of the parameter values from section S2. It implies that inactive HSC counts have a high sensitivity to changes of the rates of self-renewing divisions and activated cell differentiation (a 5% perturbation of the parameter implies a more than 40% perturbation of cell counts). Homeostatic cell counts are relatively insensitive to perturbations of all other parameters, i.e., a 5% perturbation of the respective parameter leads to less than 10% change of homeostatic cell counts. Especially, the model is

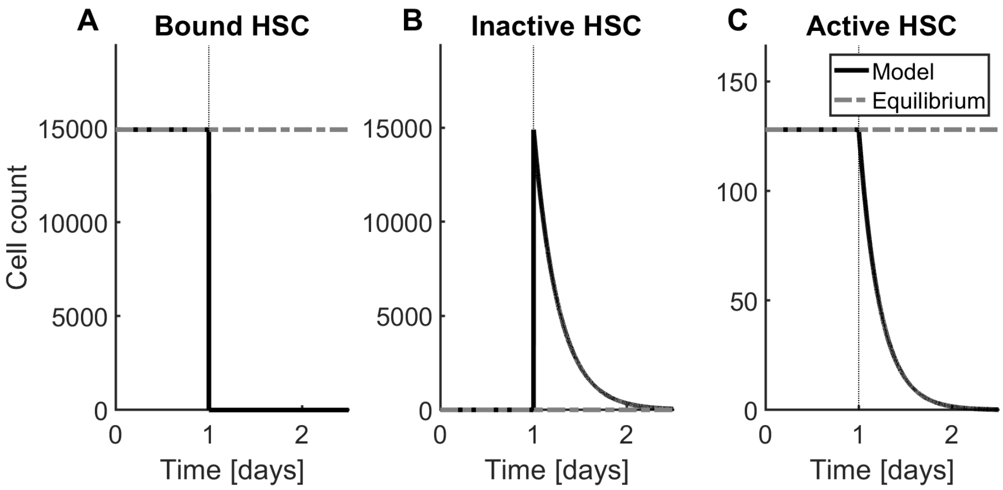

Figure S5: **System dynamics in absence of niche cells.** Starting at the equilibrium state, we set $K = 0$ at time $t = 1$ day. This corresponds to a removal of the stem cell niche. In absence of the niche stem cells become extinct due to differentiation. The dashed gray line corresponds to the homeostatic cell counts. The black lines show model dynamics in absence of the niche. A thin dotted line shows the time $t = 1$ days. Model parameters are specified in Section S2.

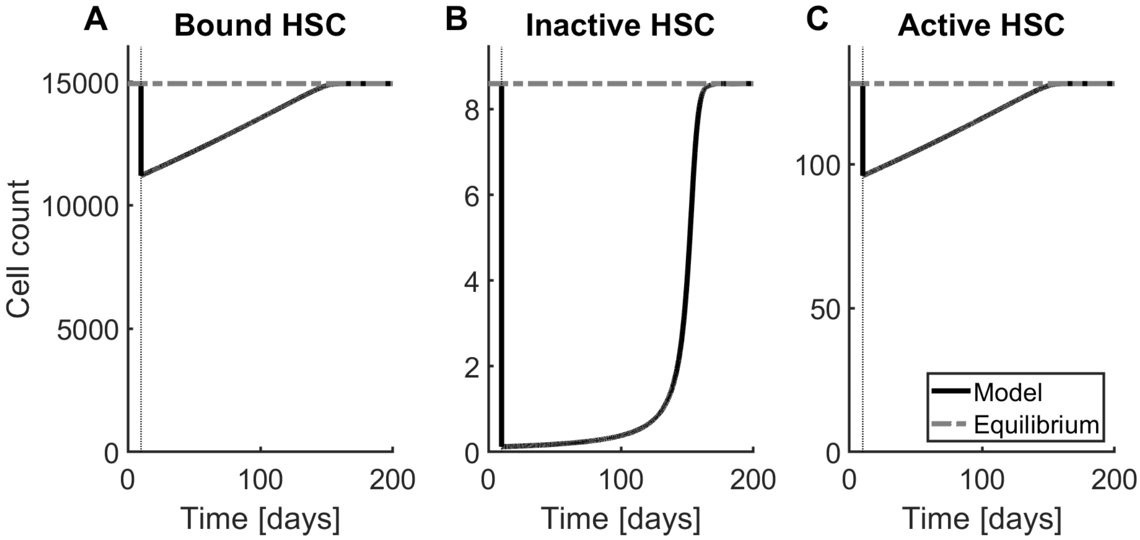

Figure S6: **System dynamics after perturbation.** Starting at the equilibrium state, 25 % of all cells are removed at time $t = 10$ days. The figure shows how the cell-counts in the model return to the equilibrium state. The dashed gray line corresponds to equilibrium cell counts. The black lines show the dynamical reaction of the system to the perturbation. A thin dotted line shows the time $t = 10$ days. Model parameters are specified in Section S2.

relatively insensitive to perturbations of the rates of attachment, detachment and inactive cell differentiation. Therefore, we consider perturbations of 100% in Figures 2-4 of the main text, where we study the transition dynamics in response to changes of these parameters.

S4 Model Analysis

S4.1 Steady states

In this section we calculate the steady states summarized in section S3.1.

The existence and uniqueness of solutions follows from the Theorem of Picard-Lindelöf.

The system (S17a) - (S17i) has three equilibria that always exist, and a fourth which only exists given certain conditions. We present all four equilibria here.

Stem cell free equilibrium

The system always has a trivial steady state

$$E_0^* := (N^*_{H,E_0}, I^*_{H,E_0}, A^*_{H,E_0}, N^*_{L,E_0}, I^*_{L,E_0}, A^*_{L,E_0})^T = (0,0,0,0,0,0)^T$$

implying $N_E^* = K$. This corresponds to the loss of all stem cells.

Monoclonal equilibria

There exist two equilibria at which one clone has become extinct and the other clone is present at positive quantities. We denote these equilibria by $E_H^* := (N^*_{H,E_H}, I^*_{H,E_H}, A^*_{H,E_H}, 0, 0, 0)^T$ and $E_L^* := (0,0,0,N^*_{L,E_L}, I^*_{L,E_L}, A^*_{L,E_L})^T$. In the following we calculate E_H^*, the expressions for E_L^* are obtained by exchanging indices H and L.

Observe that $\dot{N}_H + \dot{I}_H + \dot{A}_H = (r_H - d_{A_H})A_H - d_{I_H} I_H$. Thus, the steady states for I_H and A_H are related through

$$I_H^* = \frac{r_H - d_{A_H}}{d_{I_H}} A_H^*. \tag{S26}$$

We note that for $I_H^* > 0$ and $A_H^* > 0$ to hold, it is necessary that $r_H > d_{A_H}$. This corresponds to the biological requirement that proliferation rate of activated stem cells has to be larger than their differentiation rate. It is intuitive that this is required for maintenance of the cell population, since in a scenario where differentiation outweighs proliferation the cell number will decline to zero.

Substituting this relation in $0 = \dot{I}_H$ yields $0 = -b_H(K - N_H^*)I_H^* + 2r_H\left(\frac{d_{I_H}}{r_H - d_{A_H}}\right) I_H^* - d_{I_H} I_H^*$. Trivially, this holds for $I_H^* = 0$, which implies $A_H^* = 0$ and in turn $N_H^* = 0$. This is the stem cell free steady state.

Assuming $I_H^* \neq 0$ implies $0 = -b_H K + b_H N_H^* + \frac{2r_H d_{I_H}}{r_H - d_{A_H}} - d_{I_H}\frac{r_H - d_{A_H}}{r_H - d_{A_H}}$, which thus yields an expression for N_H in the non-trivial steady state: $N_H^* = K - \frac{d_{I_H}(r_H + d_{A_H})}{b_H(r_H - d_{A_H})}$. This allows us to find the equilibrium of activated stem cells, A_H^* since:

$$0 = \dot{A}_H \tag{S27}$$

$$0 = u_H N_H^* - r_H A_H^* - d_{A_H} A_H^* \tag{S28}$$

$$A_H^* = \frac{u_H}{r_H + d_{A_H}} N_H^* \tag{S29}$$

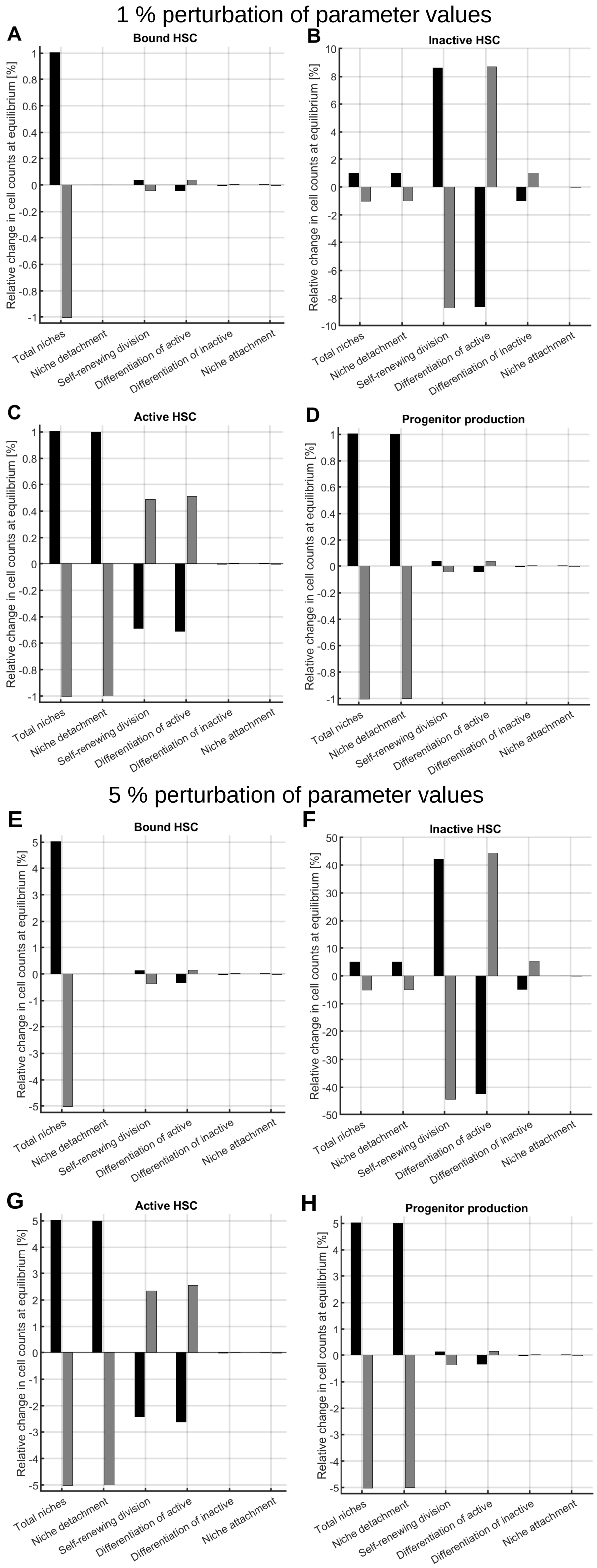

Figure S7: **Impact of niche processes on homeostatic stem cell counts.** The panels show how equilibrium stem cell counts (A-C, E-G) and progenitor production (D, H) change if a given parameter is increased or decreased by 1 % (A-D) or 5% (E-H) compared to the fitted parameters provided in Table S1. Black boxes denote an increased parameter value while gray boxes denote a decreased parameter value. Note that the impact of some parameter changes is too small to be visible on the depicted scale. The number of niche-bound stem cells is independent from the detachment rate.

The monoclonal equilibrium E_H^* with positive N_H, A_H and I_H, is then given as:

$$\begin{pmatrix} N^*_{H,E_H} \\ I^*_{H,E_H} \\ A^*_{H,E_H} \\ N^*_{L,E_H} \\ I^*_{L,E_H} \\ A^*_{L,E_H} \end{pmatrix} = \begin{pmatrix} K - \dfrac{d_{I_H}(r_H + d_{A_H})}{b_H(r_H - d_{A_H})} \\ \dfrac{r_H - d_{A_H}}{d_{I_H}} A^*_{H,E_H} \\ \dfrac{u_H}{(r_H + d_{A_H})} N^*_{H,E_H} \\ 0 \\ 0 \\ 0 \end{pmatrix} = \begin{pmatrix} K - \dfrac{d_{I_H}(r_H + d_{A_H})}{b_H(r_H - d_{A_H})} \\ \dfrac{u_H(r_H - d_{A_H})}{d_{I_H}(r_H + d_{A_H})} K - \dfrac{u_H}{b_H} \\ \dfrac{u_H}{(r_H + d_{A_H})} K - \dfrac{u_H d_{I_H}}{b_H(r_H - d_{A_H})} \\ 0 \\ 0 \\ 0 \end{pmatrix} \tag{S30}$$

Note that the number of empty niches in the steady state is simply given as $N^*_{E,E_H} = \frac{d_{I_H}(r_H + d_{A_H})}{b_H(r_H - d_{A_H})}$. This equilibrium has three positive components if and only if $r_H > d_{A_H}$ and $\frac{K b_H}{d_{I_H}} > \frac{r_H + d_{A_H}}{r_H - d_{A_H}}$.

Analogously the monoclonal equilibrium of the second clone (denoted by subscript L) has three positive components if and only if $r_L > d_{A_L}$ and $\frac{K b_L}{d_{I_L}} > \frac{r_L + d_{A_L}}{r_L - d_{A_L}}$.

Coexistence equilibria

Imposing additional conditions on the parameters, both cell clones can coexist in an equilibrium state of the system. The equilibrium will be denoted as E_B^* (with the B denoting *Both* as both clones are present).

Observe that the relation between the steady states of A_H and I_H shown in equation (S26) holds by the same argument as in the single clone case. As such we have the relations

$$I_H^* = \frac{r_H - d_{A_H}}{d_{I_H}} A_H^* \tag{S31}$$

and

$$I_L^* = \frac{r_L - d_{A_L}}{d_{I_L}} A_L^* \tag{S32}$$

Substitution of the first relation in $\dot{I}_H = 0$ yields $0 = -b_H(K - N_H^* - N_L^*) I_H^* + 2 r_H \left(\frac{d_{I_H}}{r_H - d_{A_H}}\right) I_H^* - d_{I_H} I_H^*$, implying either $I_H^* = 0$ or

$$N_E^* = K - N_H^* - N_L^* = \frac{d_{I_H}(r_H + d_{A_H})}{b_H(r_H - d_{A_H})} \tag{S33}$$

Using the analogous equation for the second clone we find that either $I_L^* = 0$ or

$$N_E^* = K - N_H^* - N_L^* = \frac{d_{I_L}(r_L + d_{A_L})}{b_L(r_L - d_{A_L})} \tag{S34}$$

In the case $I_L^* = 0$ we also have $A_L^* = 0$ due to the relation in equation (S32). This in turn implies that $N_L^* = 0$ since $\dot{N}_L = -u_L N_L$ when $I_L = 0 = A_L$. In this case we either obtain the stem cell free steady state E_0^* or the monoclonal steady state E_L^*. The analogous argumentation applies to the case $I_H^* = 0$, where we obtain either the stem cell free steady state E_0^* or the monoclonal steady state E_H^*.

Equations (S33) and (S34) imply the following necessary condition for coexistence of two clones in an equilibrium state:

$$\frac{d_{I_H}(r_H + d_{A_H})}{b_H(r_H - d_{A_H})} = \frac{d_{I_L}(r_L + d_{A_L})}{b_L(r_L - d_{A_L})} \tag{S35}$$

Notably this condition does not depend on the rates of detachment from the niche (u_H and u_L).

The condition (S35) is sufficient for existence of a manifold of coexistence equilibria. Indeed, set

$$N_E^* = \frac{d_{I_H}(r_H + d_{A_H})}{b_H(r_H - d_{A_H})} = \frac{d_{I_L}(r_L + d_{A_L})}{b_L(r_L - d_{A_L})}$$

and consider the following manifold E_B^* for $\alpha \in \mathbb{R}$. We now show the following:

For $\alpha \in (0, K - N_E^*)$ the positive coexistence equilibria are given by E_B^*.

$$E_B^* = \begin{pmatrix} N_{H,E_B}^* \\ I_{H,E_B}^* \\ A_{H,E_B}^* \\ N_{L,E_B}^* \\ I_{L,E_B}^* \\ A_{L,E_B}^* \end{pmatrix} = \begin{pmatrix} \alpha \\ \frac{u_H}{d_{I_H}} \frac{r_H - d_{A_H}}{r_H + d_{A_H}} \alpha \\ \frac{u_H}{r_H + d_{A_H}} \alpha \\ K - \alpha - N_E^* \\ \frac{u_L}{d_{I_L}} \frac{r_L - d_{A_L}}{r_L + d_{A_L}} (K - \alpha - N_E^*) \\ \frac{u_L}{r_L + d_{A_L}} (K - \alpha - N_E^*) \end{pmatrix}. \tag{S36}$$

This is the case, because for $\alpha \in \mathbb{R}$ holds that

$$\begin{aligned} \frac{d}{dt} N_H &= b_H \frac{d_{I_H}}{b_H} \frac{r_H + d_{A_H}}{r_H - d_{A_H}} \frac{r_H - d_{A_H}}{r_H + d_{A_H}} \frac{u_H}{d_{I_H}} \alpha - u_H \alpha = 0 \\ \frac{d}{dt} I_H &= -b_H \frac{d_{I_H}}{b_H} \frac{r_H + d_{A_H}}{r_H - d_{A_H}} \frac{r_H - d_{A_H}}{r_H + d_{A_H}} \frac{u_H}{d_{I_H}} \alpha + 2r_H \frac{u_H}{r_H + d_{A_H}} \alpha - d_{I_H} \frac{u_H}{d_{I_H}} \frac{r_H - d_{A_H}}{r_H + d_{A_H}} \alpha = 0 \\ \frac{d}{dt} A_H &= u_H \alpha - r_H \frac{u_H}{r_H + d_{A_H}} \alpha - d_{A_H} \frac{u_H}{r_H + d_{A_H}} \alpha = 0. \end{aligned}$$

Using $\frac{d_{I_H}(r_H + d_{A_H})}{b_H(r_H - d_{A_H})} = \frac{d_{I_L}(r_L + d_{A_L})}{b_L(r_L - d_{A_L})}$, an analogous calculation reveals that $0 = \frac{d}{dt} N_L = \frac{d}{dt} I_L = \frac{d}{dt} A_L$.

With $\alpha \in (0, K - N_E^*)$ all components of E_B^* are positive if and only if $r_H > d_{A_H}$, $r_L > d_{A_L}$ and $N_E^* < K$.

S4.2 Parameter dependence of steady state cell counts

In this section we calculate how equilibrium cell counts change under small perturbations of model parameters. This was studied numerically in section S3.4.

We can use the partial derivatives of the steady state cell counts to find the parameter-dependence of the steady state values. This method leads to the results discussed in Section 3.1 and in Table 1 of the main text.

For brevity, we omit all but one of these calculations. Apart from the following example, all the cases are straightforward.

$$\begin{aligned} \frac{\partial A_H^*}{\partial r_H} &= \frac{u_H}{r_H + d_{A_H}} \left[\left(\frac{d_{I_H}(r_H + d_{A_H})}{b_H(r_H - d_{A_H})^2} - \frac{d_{I_H}}{b_H(r_H - d_{A_H})} \right) - \frac{1}{r_H + d_{A_H}} \left(K - \frac{d_{I_H}(r_H + d_{A_H})}{b_H(r_H - d_{A_H})} \right) \right] && \text{(S37)} \\ &= \frac{u_H}{(r_H + d_{A_H})^2} \left[\left(\frac{d_{I_H}(r_H + d_{A_H})^2}{b_H(r_H - d_{A_H})^2} - \frac{d_{I_H}(r_H + d_{A_H})}{b_H(r_H - d_{A_H})} \right) - \left(K - \frac{d_{I_H}(r_H + d_{A_H})}{b_H(r_H - d_{A_H})} \right) \right] && \text{(S38)} \\ &= \frac{u_H}{(r_H + d_{A_H})^2} \left[\frac{d_{I_H}(r_H + d_{A_H})^2}{b_H(r_H - d_{A_H})^2} - K \right] && \text{(S39)} \end{aligned}$$

As such, $\frac{\partial A_H^*}{\partial r_H}$ is negative if and only if $\frac{d_{I_H}(r_H + d_{A_H})^2}{b_H(r_H - d_{A_H})^2} < K$ which is equivalent to $-\sqrt{\frac{b_H}{d_{I_H}} K} < \frac{(r_H + d_{A_H})}{(r_H - d_{A_H})} < \sqrt{\frac{b_H}{d_{I_H}} K}$. Existence of the steady state E_H^* requires that $r_H > d_{A_H}$ and $\frac{K b_H}{d_{I_H}} > \frac{r_H + d_{A_H}}{r_H - d_{A_H}}$. Since $\frac{r_H + d_{A_H}}{r_H - d_{A_H}} > 1$ the latter implies $\frac{K b_H}{d_{I_H}} > 1$.

Therefore, we obtain two biologically relevant parameter regimes, which are illustrated in Fig. S8:

- $\frac{\partial A_H^*}{\partial r_H} < 0$ if $\frac{(r_H + d_{A_H})}{(r_H - d_{A_H})} < \sqrt{\frac{b_H}{d_{I_H}} K}$, i.e., $\frac{\sqrt{\frac{b_H}{d_{I_H}} K} + 1}{\sqrt{\frac{b_H}{d_{I_H}} K} - 1} d_{AH} < r_H$
- $\frac{\partial A_H^*}{\partial r_H} > 0$ if $\sqrt{\frac{b_H}{d_{I_H}} K} < \frac{(r_H + d_{A_H})}{(r_H - d_{A_H})} < \frac{b_H}{d_{I_H}} K$

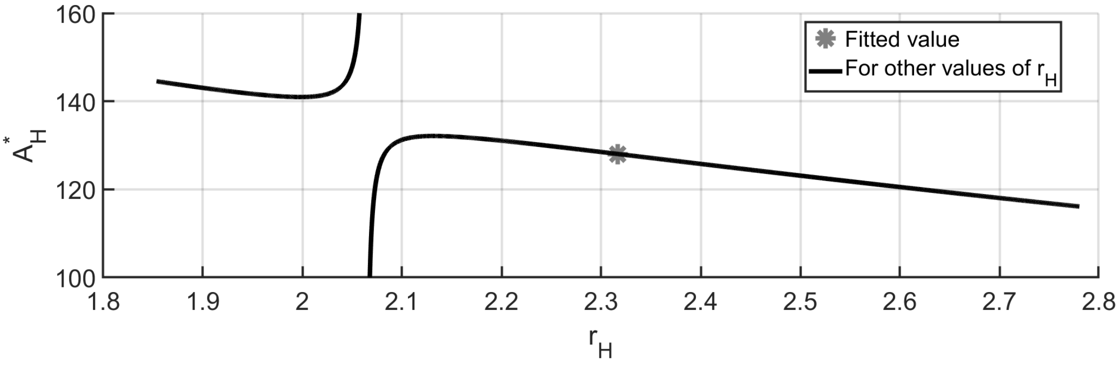

Figure S8: The dependence of the homeostatic count of activated HSC A_H^* on the parameter r_H is relatively complex. Note the singularity at $r_H = d_{A_H}$

S4.3 Parameter dependence of steady state progenitor production

In this section we calculate how equilibrium progenitor production changes under small perturbations of model parameters. This was studied numerically in section S3.4.

The production of progenitors per unit of time is described in the model by

$$i_{D_H} = 2d_{A_H}A_H + d_{I_H}I_H \tag{S40}$$

In the steady state situation, where $\frac{dI_H}{dt} = \frac{dA_H}{dt} = 0$, the production of progenitors should be in an equilibrium with the outflux of progenitors due to mature cell production. Hence determining how the steady state production of progenitors is influenced by changes of models parameters gives insights about the effect of the respective parameter changes on steady state mature cell production. In the following we study how perturbations of the parameters impact the steady state production of progenitors. The steady state production of progenitors is $i_{D_H}^* = 2d_{A_H}A_H^* + d_{I_H}I_H^*$.

In supplementary section S4.1 we showed that in general, $I_H^* = \frac{r_H - d_{A_H}}{d_{I_H}}A_H^*$ and $A_H^* = \frac{u_H}{r_H + d_{A_H}}N_H^*$. Therefore,

$$i_{D_H}^* = \left(2d_{A_H} + d_{I_H}\frac{r_H - d_{A_H}}{d_{I_H}}\right)A_H^* \tag{S41}$$

$$= (r_H + d_{A_H})\,A_H^* \tag{S42}$$

$$= \frac{r_H + d_{A_H}}{r_H + d_{A_H}}u_H N_H^* = u_H N_H^* \tag{S43}$$

Thus, in steady state, the production of progenitors scales with $u_H N_H^*$. Calculating the partial derivatives of $u_H N_H^*$ with respect to the parameters and assuming $N_H^* > 0$ reveals the dependence on parameter perturbations discussed in the main text: $i_{D_H}^*$ increases with K, u_H, r_H and b_H, but decreases when d_{A_H} or d_{I_H} increases. In other words, increasing differentiation of HSC decreases the production of progenitors and in turn mature blood cells.

S4.4 Stability analysis

Linear stability of the homeostatic state

In this section we mathematically show the linear asymptotic stability of the homeostatic equilibrium in absence of mutated cells. It was studied numerically in section S3.3.

Proposition: In absence of a second clone the homeostatic steady state is linearly stable whenever it exists i.e., if $r_H > d_{A_H}$, $\dfrac{d_{I_H}(r_H + d_{A_H})}{b_H(r_H - d_{A_H})} < K$.

Proof: Consider the linearization of model for one HSC clone around the homeostatic steady state, E_H^*:

$$A_1 = \begin{pmatrix} -b_H I_H^* - u_H & b_H N_{E,E_H}^* & 0 \\ b_H I_H^* & -b_H N_{E,E_H}^* - d_{I_H} & 2r_H \\ u_H & 0 & -r_H - d_{A_H} \end{pmatrix} \tag{S44}$$

The characteristic polynomial of A_1 is:

$$\chi_{A_1}(X) = \det(A_1 - \mathbb{1}_{3\times 3}X) \tag{S45}$$

$$= (-b_H I_H^* - u_H - X)(-b_H N_{E,E_H}^* - d_{I_H} - X)(-r_H - d_{A_H} - X) - b_H N_{E,E_H}^* \left[b_H I_H^*(-r_H - d_{A_H} - X) - 2r_H u_H\right] \tag{S46}$$

which can be written on the form $\chi_{A_1}(X) = X^3 + a_2X^2 + a_1X + a_0$, with factors

$$a_2 = b_H \left(I_H^* + N_{E,E_H}^*\right) + d_{A_H} + d_{I_H} + r_H + u_H \tag{S47}$$

$$a_1 = b_H(r_H + d_{A_H})\left(I_H^* + N_{E,E_H}^*\right) + b_H d_{I_H} I_H^* + b_H u_H N_{E,E_H}^* + u_H(d_{A_H} + d_{I_H} + r_H) + d_{A_H} d_{I_H} + r_H d_{I_H} \tag{S48}$$

$$a_0 = d_{I_H}(r_H + d_{A_H})(b_H I_H^* + u_H) - b_H u_H (r_H - d_{A_H}) N_{E,E_H}^* = 3u_H d_{I_H}(r_H + d_{A_H}) \tag{S49}$$

The Routh-Hurwitz Criterion ensures that the polynomial only has roots with negative real parts if

(i) $a_2 > 0$ and

(ii) $a_2a_1 - a_0 > 0$ and

(iii) $a_0 > 0$.

(i) and *(iii)* are fulfilled. *(ii)* is a consequence of the following calculation.

$$\begin{aligned}
&- a_0 + a_2a_1 \\
=&- 3u_H d_{I_H}(r_H + d_{A_H}) + \Big[\underbrace{\Big(b_H\left(I_H^* + N_{E,E_H}^*\right)\Big)}_{=:\xi_1>0} + \underbrace{\Big(r_H + d_{A_H} + d_{I_H} + u_H\Big)}_{=:\xi_2>0}\Big]\times \\
&\Big[\underbrace{\Big(b_H(r_H + d_{A_H})\left(I_H^* + N_{E,E_H}^*\right) + b_H d_{I_H} I_H^* + b_H u_H N_{E,E_H}^*\Big)}_{=:\xi_3>0} + \underbrace{\Big(u_H(d_{A_H} + d_{I_H} + r_H) + d_{I_H}(d_{A_H} + r_H)\Big)}_{=:\xi_4>0}\Big] \\
=&- 3u_H d_{I_H}(r_H + d_{A_H}) + \xi_1\xi_3 + \xi_1\xi_4 + \xi_2\xi_3 + \Big((r_H + d_{A_H}) + d_{I_H} + u_H\Big)\Big(u_H(d_{A_H} + r_H) + u_H d_{I_H} + d_{I_H}(d_{A_H} + r_H)\Big) \\
=&- 3u_H d_{I_H}(r_H + d_{A_H}) + u_H(r_H + d_{A_H})^2 + u_H d_{I_H}(r_H + d_{A_H}) + d_{I_H}(r_H + d_{A_H})^2 + u_H d_{I_H}(r_H + d_{A_H}) + u_H d_{I_H}^2 \\
&+ d_{I_H}^2(r_H + d_{A_H}) + u_H^2(r_H + d_{A_H}) + u_H^2 d_{I_H} + u_H d_{I_H}(r_H + d_{A_H}) \\
=&u_H(r_H + d_{A_H})^2 + d_{I_H}(r_H + d_{A_H})^2 + u_H d_{I_H}^2 + d_{I_H}^2(r_H + d_{A_H}) + u_H^2(r_H + d_{A_H}) + u_H^2 d_{I_H} \\
>&0
\end{aligned}$$

Hence, all roots of the polynomial $\chi_{A_1}(X)$ have negative real parts. Thus, the eigenvalues of A_1 all have negative real part and the steady state E_H^* is locally stable.

□

Model dynamics in presence of one clone are summarized in Figure S9.

Perturbations of the homeostatic state and clonal fitness

In this section we investigate under which conditions the healthy equilibrium can be destabilized by adding a second stem cell clone. Based on this we derive the expressions for the clonal fitness.

We assume that $r_H > D_{A_H}$, $r_L > d_{A_L}$, $\dfrac{d_{I_H}(r_H + d_{A_H})}{b_H(r_H - d_{A_H})} < K$, and $\dfrac{d_{I_L}(r_L + d_{A_L})}{b_L(r_L - d_{A_L})} < K$ which is necessary and sufficient for existence of the two monoclonal steady states E_H^* and E_L^*.

In the following we show that if we add a small number of malignant cells to the homeostatic state these cells will expand if $\dfrac{d_{I_H}(r_H + d_{A_H})}{b_H(r_H - d_{A_H})} > \dfrac{d_{I_L}(r_L + d_{A_L})}{b_L(r_L - d_{A_L})}$ and die out if $\dfrac{d_{I_H}(r_H + d_{A_H})}{b_H(r_H - d_{A_H})} < \dfrac{d_{I_L}(r_L + d_{A_L})}{b_L(r_L - d_{A_L})}$.

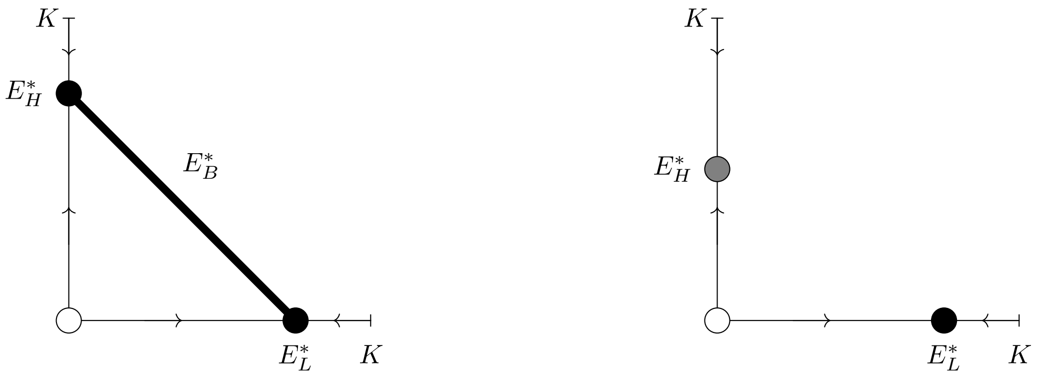

(a) (b)

Figure S9: **Phase plane illustrations of equilibria.** The circles and the thick black line denote equilibria. The white circle corresponds to the stem cell free equilibrium E_0^*. E_H^* and E_L^* correspond to monoclonal equilibria. The thick line E_B^* corresponds to the manifold of equilibria where two clones coexist. The arrows depict system dynamics in the presence of a single clone. If both clones have equal fitness, the system will move asymptotically toward a point on the thick line, which depends on the initial conditions. In panel a the two clones have equal fitness, whereas panel b depicts a scenario where the mutated clone (denoted by L) has a competitive advantage.

Proposition: Consider the model (S17a)-(S17i) of two competing clones. Let $r_H > d_{A_H}$, $r_L > d_{A_L}$, $\dfrac{d_{I_H}(r_H + d_{A_H})}{b_H(r_H - d_{A_H})} < K$, and $\dfrac{d_{I_L}(r_L + d_{A_L})}{b_L(r_L - d_{A_L})} < K$. The homeostatic steady state is linearly asymptotically stable if and only if $\dfrac{d_{I_H}(r_H + d_{A_H})}{b_H(r_H - d_{A_H})} > \dfrac{d_{I_L}(r_L + d_{A_L})}{b_L(r_L - d_{A_L})}$.

Proof: Consider the linearization of the model around the healthy equilibrium.

The linearization of the model around the homeostatic equilibrium E_H^* is given by

$$A = \begin{pmatrix} A_1 & A_2 \\ 0 & A_4 \end{pmatrix},$$

where A_1 is the linarization of the model for one HSC clone around the homeostatic steady state, A_2 is a real 3×3 matrix and

$$A_4 = \begin{pmatrix} -u_L & b_L N_{E,E_H}^* & 0 \\ 0 & -b_L N_{E,E_H}^* - d_{I_L} & 2r_L \\ u_L & 0 & -r_L - d_{A_L} \end{pmatrix}. \tag{S50}$$

The characteristic polynomial of A is

$$\chi_A(X) = \det(A - \mathbb{1}_{6\times 6} X) \tag{S51}$$

$$= \left| \begin{pmatrix} A_1 - \mathbb{1}_{3\times 3} X & A_2 \\ 0 & A_4 - \mathbb{1}_{3\times 3} X \end{pmatrix} \right| \tag{S52}$$

$$= \chi_{A_1}(X) \cdot \chi_{A_4}(X). \tag{S53}$$

The latter holds since A is a block triangular matrix. The eigenvalues of A_1 correspond to the eigenvalues of the linearization of the model for one HSC clone around the homeostatic steady state. We know from the

previous section that they are negative. We now have to investigate the sign of the real parts of the roots of $\chi_{A_4}(X)$. It holds

$$\chi_{A_4}(X) = \left| \begin{pmatrix} X+u_L & -b_L N^*_{E,E_H} & 0 \\ 0 & X+b_L N^*_{E,E_H}+d_{I_L} & -2r_L \\ -u_L & 0 & X+r_L+d_{A_L} \end{pmatrix} \right| \tag{S54}$$

$$= (X+u_L)(X+b_L N^*_{E,E_H}+d_{I_L})(X+r_L+d_{A_L}) - 2r_L u_L b_L N^*_{E,E_H} \tag{S55}$$

We note that

$$\det(a_4) = \chi_{A_4}(0) = u_L(b_L N^*_{E,E_H}+d_{I_L})(r_L+d_{A_L}) - 2r_L u_L b_L N^*_{E,E_H} \tag{S56}$$

$$= u_L(b_L N^*_{E,E_H} r_L + d_{I_L} r_L + b_L N^*_{E,E_H} d_{A_L} + d_{I_L} d_{A_L}) - 2r_L u_L b_L N^*_{E,E_H} \tag{S57}$$

$$= u_L(-b_L N^*_{E,E_H} r_L + d_{I_L} r_L + b_L N^*_{E,E_H} d_{A_L} + d_{I_L} d_{A_L}) \tag{S58}$$

$$= u_L\big(b_L N^*_{E,E_H}(d_{A_L}-r_L) + d_{I_L}(d_{A_L}+r_L)\big). \tag{S59}$$

Furthermore $\det(A_4) > 0 \Leftrightarrow N^*_{E,E_H} < \frac{d_{I_L}(r_L+d_{A_L})}{b_L(r_L-d_{A_L})}$. Together with the expression for N^*_{E,E_H}, we obtain

$$\det(A_4) > 0 \Leftrightarrow \frac{d_{I_H}(r_H+d_{A_H})}{b_H(r_H-d_{A_H})} < \frac{d_{I_L}(r_L+d_{A_L})}{b_L(r_L-d_{A_L})}.$$

The characteristic polynomial expands to

$$\chi_{A_4}(X) = X^3 + (u_L + b_L N^*_{E,E_H} + d_{I_L} + r_L + d_{A_L})X^2 \tag{S60}$$

$$+ [u_L(b_L N^*_{E,E_H} + d_{I_L} + r_L + d_{A_L}) + (r_L+d_{A_L})(b_L N^*_{E,E_H} + d_{I_L})]X \tag{S61}$$

$$+ u_L\big(b_L N^*_{E,E_H}(d_{A_L}-r_L) + d_{I_L}(d_{A_L}+r_L)\big) \tag{S62}$$

We now check the Routh-Hurwitz Criterion. We write $\chi_{A_4}(X) := X^3 + a_2X^2 + a_1X + a_0$. The polynomial has only roots with negative real parts if

(i) $a_2 > 0$ and

(ii) $a_2a_1 - a_0 > 0$ and

(iii) $a_0 > 0$.

(i) is satisfied, since it is a sum of positive terms. (ii) expands to

$$[u_L(b_L N^*_{E,E_H} + d_{I_L} + r_L + d_{A_L}) + (r_L+d_{A_L})(b_L N^*_{E,E_H} + d_{I_L})][u_L + b_L N^*_{E,E_H} + d_{I_L} + r_L + d_{A_L}] \tag{S63}$$

$$- u_L b_L N^*_{E,E_H} d_{A_L} + u_L r_L - u_L d_{I_L}(d_{A_L}+r_L) \tag{S64}$$

$$=[u_L(b_L N^*_{E,E_H} + d_{I_L} + r_L + d_{A_L}) + (r_L+d_{A_L})(b_L N^*_{E,E_H} + d_{I_L})][b_L N^*_{E,E_H} + d_{I_L} + r_L + d_{A_L}] \tag{S65}$$

$$+ u_L[u_L(b_L N^*_{E,E_H} + d_{I_L} + r_L + d_{A_L}) + (r_L+d_{A_L})(b_L N^*_{E,E_H} + d_{I_L})] \tag{S66}$$

$$- u_L b_L N^*_{E,E_H} d_{A_L} + u_L r_L - u_L d_{I_L}(d_{A_L}+r_L) \tag{S67}$$

$$=[u_L(b_L N^*_{E,E_H} + d_{I_L} + r_L + d_{A_L}) + (r_L+d_{A_L})(b_L N^*_{E,E_H} + d_{I_L})][b_L N^*_{E,E_H} + d_{I_L} + r_L + d_{A_L}] \tag{S68}$$

$$+ u_L[u_L(b_L N^*_{E,E_H} + d_{I_L} + r_L + d_{A_L})] + u_L(r_L+d_{A_L})b_L N^*_{E,E_H} + u_L(r_L+d_{A_L})d_{I_L} \tag{S69}$$

$$- u_L b_L N^*_{E,E_H} d_{A_L} + u_L r_L - u_L d_{I_L}(d_{A_L}+r_L) \tag{S70}$$

$$=[u_L(b_L N^*_{E,E_H} + d_{I_L} + r_L + d_{A_L}) + (r_L+d_{A_L})(b_L N^*_{E,E_H} + d_{I_L})][b_L N^*_{E,E_H} + d_{I_L} + r_L + d_{A_L}] \tag{S71}$$

$$+ u_L[u_L(b_L N^*_{E,E_H} + d_{I_L} + r_L + d_{A_L})] + u_L r_L b_L N^*_{E,E_H} + u_L r_L \tag{S72}$$

which is a sum of positive terms and hence positive. As shown above (iii) holds if and only if

$$\frac{d_{I_H}(r_H+d_{A_H})}{b_H(r_H-d_{A_H})} < \frac{d_{I_L}(r_L+d_{A_L})}{b_L(r_L-d_{A_L})}$$

□

Taken together this implies that the homeostatic state cannot be destabilized by a second clone if

$$\frac{d_{I_H}(r_H + d_{A_H})}{b_H(r_H - d_{A_H})} < \frac{d_{I_L}(r_L + d_{A_L})}{b_L(r_L - d_{A_L})}.$$

However in the opposite case $\frac{d_{I_H}(r_H + d_{A_H})}{b_H(r_H - d_{A_H})} > \frac{d_{I_L}(r_L + d_{A_L})}{b_L(r_L - d_{A_L})}$ the homeostatic state is destabilized. In the first case, leukemic cells introduced to the system die out whereas in the second case they grow. This implies that the relation between $\frac{d_{I_L}(r_L+d_{A_L})}{b_L(r_L-d_{A_L})}$ and $\frac{d_{I_H}(r_H+d_{A_H})}{b_H(r_H-d_{A_H})}$ decides whether an additional clone introduced in the system can persist or not.

These findings provide the theoretical foundation for the following notion of *fitness*. If we define the fitness of the HSC by $F_H := \frac{b_H(r_H - d_{A_H})}{d_{I_H}(r_H + d_{A_H})}$ and the fitness of LSC by $F_L := \frac{b_L(r_L - d_{A_L})}{d_{I_L}(r_L + d_{A_L})}$, then the clone with the larger fitness can destabilize the homeostatic state of the clone with lower fitness but not vice versa.

S5 Peripheral blood malignant cell burden can change faster or slower compared to the malignant cell burden in the stem cell niche

Simulations indicate that the malignant cell burden in the stem cell compartment and in the peripheral blood can differ significantly. Figure S10a provides an example for a scenario where the malignant cell burden in the stem cell niche increases earlier and is higher compared to the progenitor compartment, while figure S10b provides an example for a scenario where the malignant cell burden increases earlier and is higher in the progenitor compartment compared to the stem cell compartment.

S6 Multiple stem cell doses increase chimerism in the unpreconditioned host but not in the preconditioned host

To reduce complications resulting from low circulating blood cell counts after bone marrow transplantation different approaches have been suggested. One approach involves splitting the transplant in multiple doses that are infused on subsequent days [4, 22]. In unpreconditioned mice the latter results in a higher chimerism [4]. This observation supports the concept that there exist empty niches under steady state conditions that are filled by subsequent transplantation of small cell doses [4]. Our calibrated model is able to reproduce this observation as discussed in supplementary section S2.

It is a clinically relevant question, whether the engraftment of transplanted cells in humans can be improved if the transplant is split into multiple doses. This question not only arises in the context of bone marrow transplantation in the case of hematologic malignancy, it is also relevant when genetically engineered HSC are used to cure metabolic diseases [27, 2, 16]. Furthermore, a transplant splitting approach could be useful if it is combined with a reduced intensity conditioning (RIC) regime. This could allow to further reduce the complications of stem cell transplants in the elderly.

To obtain insights into the question how preconditioning affects the advantageous impact of splitting a transplant into multiple doses, we simulate the following scenario: Starting at homeostasis, one third of the niche-bound HSC were replaced by a leukemic clone with competitive advantage. This scenario corresponds to the setting of a newly diagnosed acute myeloid leukemia (AML) patient. Preconditioning was modeled as pre-transplant ablation of a prescribed fraction of healthy and leukemic cells. We then simulated transplantation of healthy HSC either as single dose or as multiple subsequent doses adding up to the same cell-count. To evaluate the outcome, we use the residual disease 100 days after transplantation.

The number of transplanted healthy HSC were chosen as $3.75 \cdot 10^4$ for the first dose and $2 \cdot 10^4$ for the subsequent doses to mirror the methodology of Landau et al. [22]. Note that this is two orders of magnitude lower than the cell-counts described by Landau et al. [22], since we do not assume all transplanted cells be to HSC.

The results are shown in Figure S11. Our simulations indicate that the benefit of splitting a transplant into multiple doses is greater for the unpreconditioned host. In the unpreconditioned host a reduction in the residual disease at day 100 of up to 8% was achieved when the transplant was split in multiple smaller doses. However, with preconditioning this effect becomes negligible. This finding is in line with results from a clinical trial [22].

The model suggests that subsequent transplantation of multiple cell doses could be beneficial compared to a single dose transplant only in absence of relevant preconditioning.

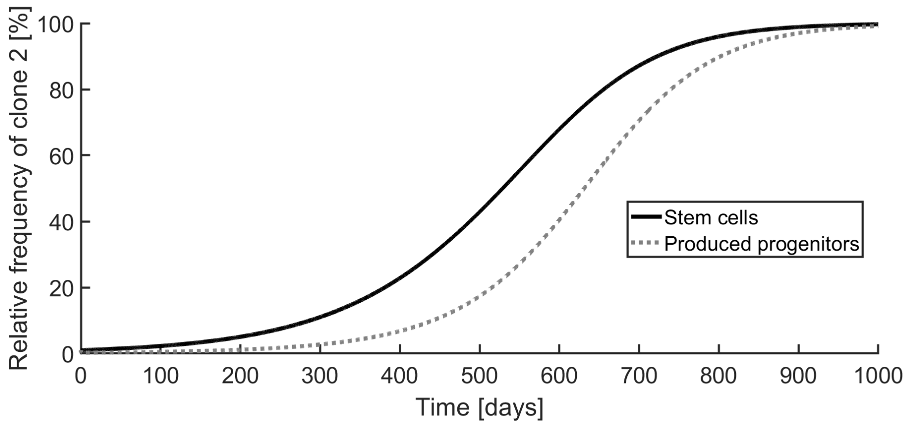

(a)

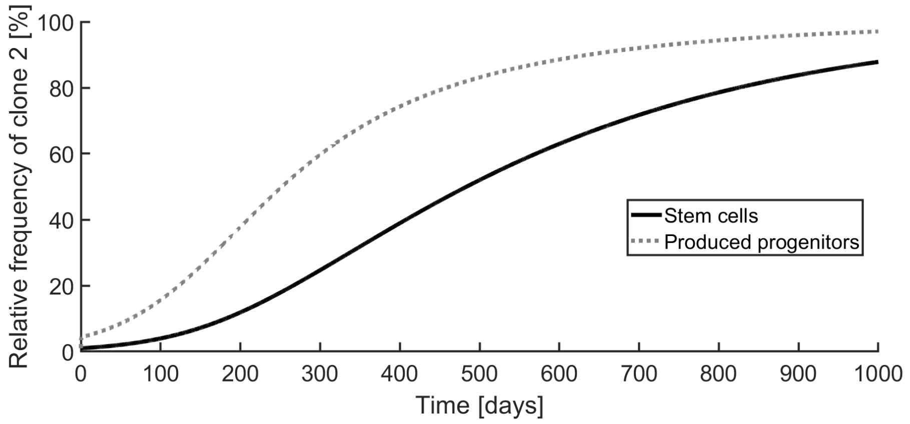

(b)

Figure S10: **Time evolution of the malignant cell burden in the stem and progenitor compartment.** In both panels, the niche is invaded by a clone with a fitness advantage due to a higher proliferation rate (referred to as clone 2). In panel (b) the advantage is smaller compared to panel (a). In addition in the scenario depicted in panel (a) the invading clone has a decreased detachment rate compared to the wild-type clone, while in panel (b) the detachment rate of the invading clone is increased compared to the wild-type cells. The simulations show that depending on stem cell parameters the malignant cell burden in the progenitor compartment can increase before or after the malignant cell burden in the stem cell compartment. Parameters: (a), $r_2 = 1.2r_1$ and $u_2 = 5u_1$; (b) $r_2 = 4r_1$ and $u_2 = 0.4u_1$. The remaining parameters are as in Table S1.

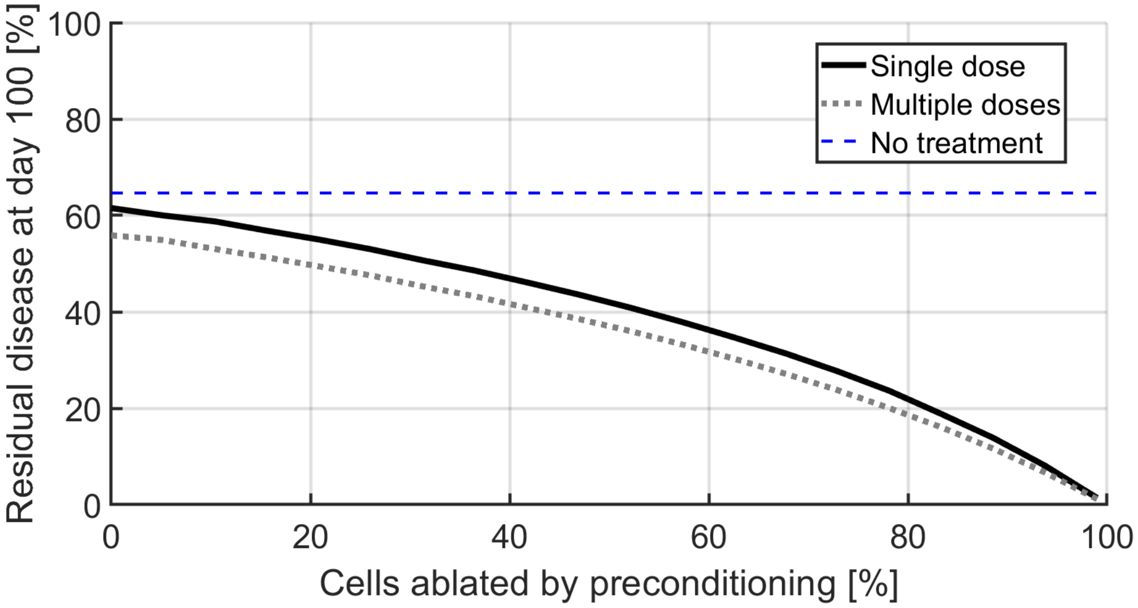

Figure S11: **Residual disease after bone marrow transplantation with and without preconditioning.** Before preconditioning, 33% of the stem cell niche are occupied by a leukemic clone with a competitive advantage. Without treatment, the leukemic cell mass increases to approx. 65% within 100 days, shown as a dashed blue line. Before stem cells are transplanted a variable fraction of leukemic and healthy cells is ablated due to preconditioning. Preconditioning is assumed to be equally effective for healthy and malignant stem cells. To model the transplant either one dose of free healthy stem cells or multiple doses at subsequent days were added to the system. The total amount of transplanted cells is equal in both cases. The cell counts were based on the experimental work of Landau et al. [22] as described in the text. The effect of splitting the transplant in multiple doses decreases in case of preconditioning.

S7 Mobilizing agents could decrease the residual disease in the unpreconditioned host but lead to an increase in the preconditioned host.

As shown in the previous section, our simulations indicate that the chimerism obtained after stem cell transplantation to an unpreconditioned host crucially depends on the amount of free niche spaces. This finding led us to question how the chimerism could be affected by stem cell mobilizing agents.

Mobilization agents force stem cells to detach from the niche. This could improve the efficiency of a bone marrow transplant following reduced or no preconditioning. One question arising in this context is whether no or reduced intensity conditioning (RIC) accompanied by mobilization agents and stem cell transplantation could be a treatment option for hematological cancers in the elderly not eligible for conventional transplantation regimens that require high dose chemotherapy. On the one hand, the use of mobilization agents could increase the number of donor cells homing to the niche and thus reduce the malignant cell burden. On the other hand detached stem cells are more prone to proliferate [34, 32, 33, 46]. For this reason stem cell mobilization could trigger the expansion of the malignant cell population. To better understand the relative contribution of these two processes to disease evolution we simulated a scenario where a malignant stem cell clone with a competitive advantage initially occupies one third of the bone marrow niche. First we consider a scenario without preconditioning. A transplant of healthy stem cells is introduced at day 0 and the detachment rate of healthy and malignant stem cells is increased at the day of transplantation due to mobilization agents. Later the detachment rate returns to its original value. The model was simulated using the parameters and transplant doses given in section S2 of this supplement. We assume that mobilizing agents act immediately when administered. This is a good approximation of reality, since the peak effect of mobilizing agents is reached after 8-12 hours [30]. This is short compared to the pre-clinical phase of malignant diseases which takes multiple years [13]. Similarly, we neglect the time transplanted cells require to home to the bone marrow, which is approximately one day [4, 15].

The simulations suggest that stem cell mobilization for short periods of time leads to an increased engraftment of healthy cells compared to the setting without mobilization. However, the number of malignant cells increases if the duration of mobilization treatment is longer than a critical time t_c. The stronger the mobilization effect, the earlier the malignant cell expansion dominates and the smaller the value of t_c. Figure S12 shows after which duration of mobilization treatment the malignant cell burden starts to increase and how this depends on the strength of the mobilization effect.

To consider the effect of mobilizing agents combined with preconditioning, we simulated the same scenario as above, with a malignant stem cell subpopulation occupying one third of the stem cell niche. On day 0, preconditioning was simulated by removing a certain percentage of all stem cells. We assume that preconditioning acts equally on healthy and malignant stem cells. After preconditioning, a transplant of free healthy cells was added, accompanied by mobilizing agents that were effective for 0.5 days.

In figure S13 we show the changes to the residual disease after 20 days in comparison to the same scenario without mobilization. The simulations suggest that following efficient preconditioning (i.e. preconditioning that ablates large numbers of stem cells), increased mobilization leads to an increase in residual disease. However, if preconditioning is performed with reduced intensity, the increased mobilization can lead to a decrease in residual disease.

The effect of mobilizing agents may also be of relevance when genetically engineered cells have to be introduced to the stem cell niche of an unpreconditioned host, as in the case of gene therapy. Next we addressed the question whether the number of genetically engineered cells homing to the bone marrow could be increased by using mobilizing agents prior to transplantation. Figure S14 shows that in case of no preconditioning the number of genetically engineered stem cells homing to the niche increases if mobilizing agents are administered before transplantation, however this effect becomes negligible in the context of preconditioning.

Taken together our simulations suggest that mobilizing agents could improve the homing of transplanted cells to the niche only in hosts with reduced or no preconditioning.

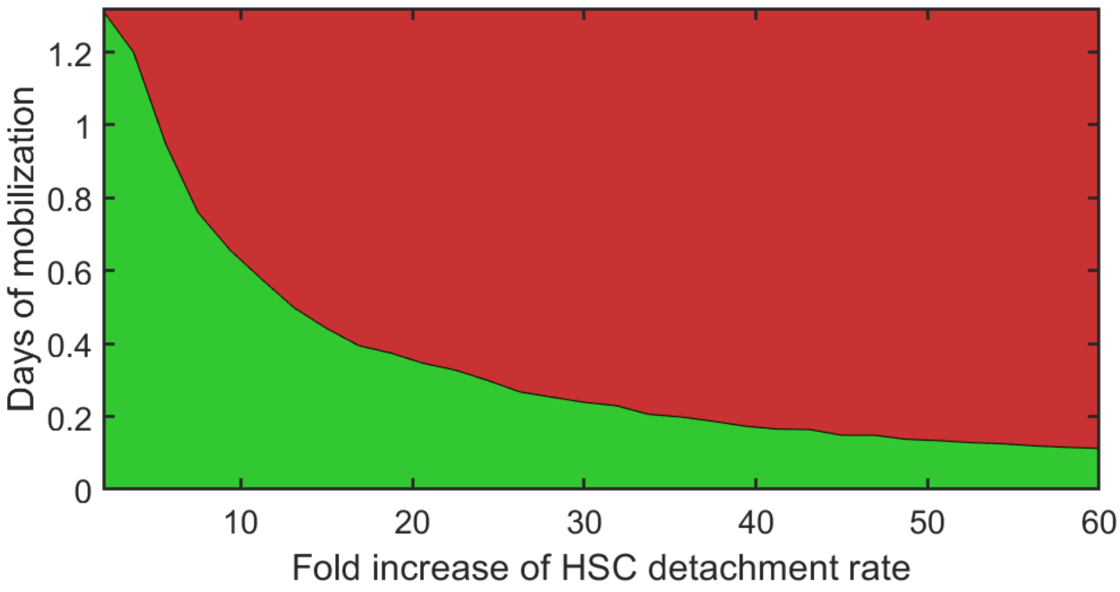

Figure S12: **Impact of mobilizing agents on the non-preconditioned host.** In an unpreconditioned host stem cell mobilizing agents can increase the number of graft-related cells homing to the niche. This allows for a reduction of the malignant cell burden in absence of preconditioning. However, if the effect of the mobilizing agents lasts longer than a critical time t_c, malignant cells detached from the niche expand and increase the disease burden. To assess this effect, we compare the following two hypothetical therapy approaches: (i) bone marrow transplantation without preconditioning, (ii) bone marrow transplantation without preconditioning accompanied by mobilizing agents for up to 1.3 days. We compare the malignant cell burden after 100 days. When the disease burden after strategy (ii) is not higher compared to strategy (i) we define the effect of mobilization as beneficial. This is indicated by the green area. At the beginning of the simulations a leukemic clone occupies one third of the niche. The horizontal axis quantifies by how much the mobilizing agent increases the rate of stem cell detachment from the niche relative to wild type HSC, the vertical axis quantifies how long the mobilizing effect remains in the system.

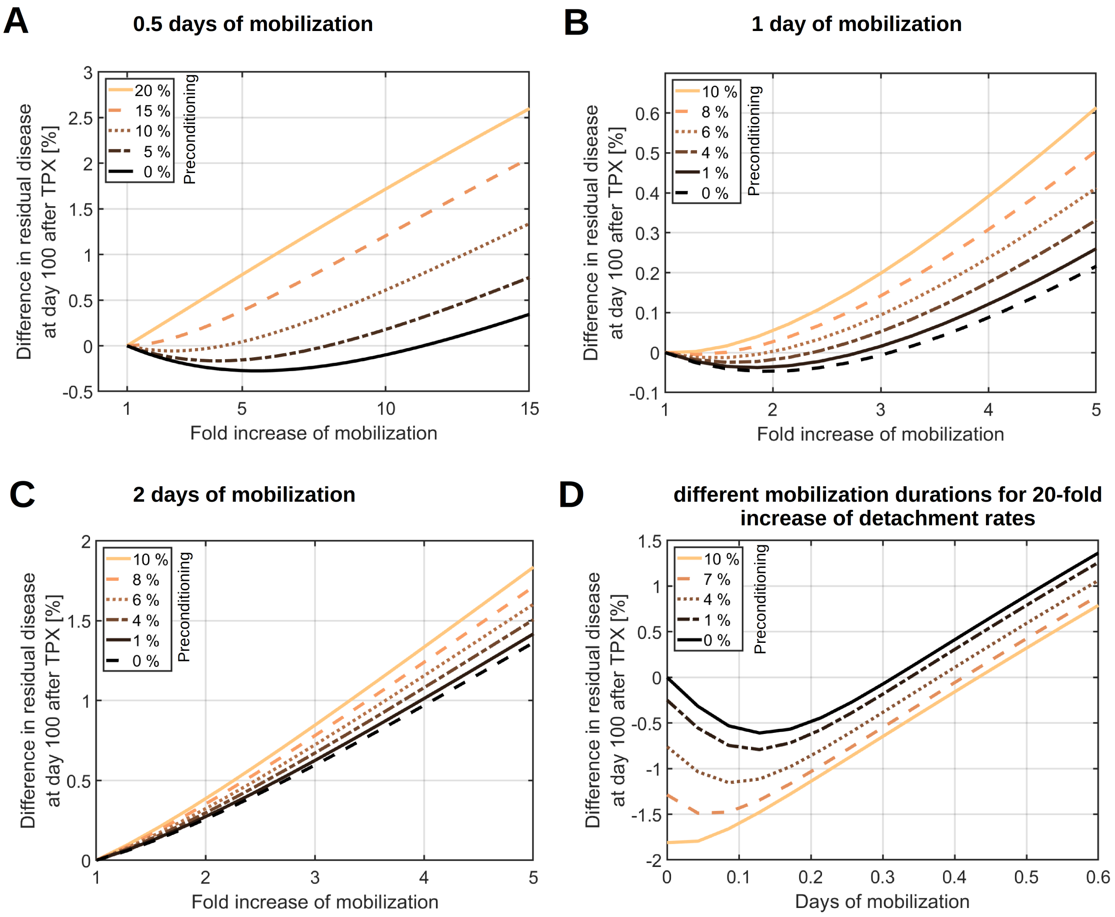

Figure S13: **Impact of mobilizing agents after preconditioning.** Before transplantation a fraction of HSC was ablated by preconditioning ("0%" indicates no preconditioning, "20%" indicates ablation of 20% of healthy and malignant stem cells from the niche.) After ablation an HSC transplant was added. Post transplantation HSC detachment was increased by mobilizing agents for 0.5 days (A), 1 day (B) or 2 days (C). The x-axis quantifies by which factor mobilization agents increase the HSC detachment rate. The y-axis shows the increase of malignant cells due to the mobilizing agent (residual disease after therapy with preconditioning, transplantation plus mobilization minus residual disease after therapy with preconditioning and transplantation). Negative values indicate that the mobilizing agent has a beneficial effect. For two days of mobilization there is no beneficial effect, regardless of the strength of the mobilization. Preconditioning of 0% is also shown in Fig. S12. The curve for 0% preconditioning in (A) corresponds to a horizontal line in Fig. S12 intersecting the y-axis at 0.5 days. Red color in Fig. S12 corresponds to positive values in (A), green color to negative values. Analogous relations hold for (B) and (C). In (D) the HSC detachment rate is increased by a factor of 20 for the duration indicated on the x-axis. The y-axis shows therapy outcome after 100 days. The preconditioning of 0% in (D) corresponds to a vertical line in Fig. S12 intersecting the x-axis at 20. Again, negative values correspond to green, positive to red color in Fig. S12.

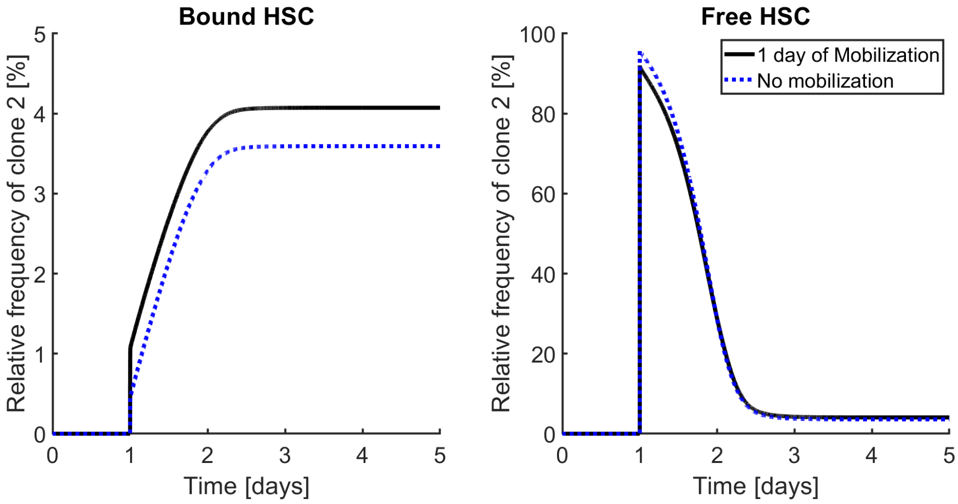

Figure S14: **Impact of mobilizing agents on the homing of genetically engineered HSC in unpreconditioned hosts.** In the non-preconditioned host, the number of genetically engineered stem cells (referred to as clone 2) homing to the bone marrow can be increased by the use of mobilizing agents prior to transplantation. In the simulation depicted in black HSC mobilization has been increased by a factor of 2 during the day before transplantation, which is a standard duration in clinical practice [35]. At the time of transplantation, the drug-induced mobilization was assumed to have stopped. For comparison, the blue dotted lines depicts a scenario with no increase in HSC mobilization.

References

[1] Abkowitz, J. L., Catlin, S. N., McCallie, M. T. and Guttorp, P. [2002], 'Evidence that the number of hematopoietic stem cells per animal is conserved in mammals', *Blood* **100**(7), 2665–2667.

[2] Aiuti, A., Slavin, S., Aker, M., Ficara, F., Deola, S., Mortellaro, A., Morecki, S., Andolfi, G., Tabucchi, A., Carlucci, F., Marinello, E., Cattaneo, F., Vai, S., Servida, P., Miniero, R., Roncarolo, M. G. and Bordignon, C. [2002], 'Correction of ADA-SCID by stem cell gene therapy combined with nonmyeloablative conditioning.', *Science* **296**(5577), 2410–2413.

[3] Ashcroft, P., Manz, M. G. and Bonhoeffer, S. [2017], 'Clonal dominance and transplantation dynamics in hematopoietic stem cell compartments', *PLOS Computational Biology* **13**(10), e1005803.

[4] Bhattacharya, D., Czechowicz, A., Ooi, A. L., Rossi, D. J., Bryder, D. and Weissman, I. L. [2009], 'Niche recycling through division-independent egress of hematopoietic stem cells', *J Exp Med* **206**(12), 2837–2850.

[5] Bhattacharya, D., Rossi, D. J., Bryder, D. and Weissman, I. L. [2006], 'Purified hematopoietic stem cell engraftment of rare niches corrects severe lymphoid deficiencies without host conditioning', *J Exp Med* **203**(1), 73–85.

[6] Boyd, A. L., Campbell, C. J. V., Hopkins, C. I., Fiebig-Comyn, A., Russell, J., Ulemek, J., Foley, R., Leber, B., Xenocostas, A., Collins, T. J. and Bhatia, M. [2014], 'Niche displacement of human leukemic stem cells uniquely allows their competitive replacement with healthy HSPCs.', *J Exp Med* **211**(10), 1925–1935.

[7] Buccisano, F., Maurillo, L., Schuurhuis, G., Del Principe, M., Di Veroli, A., Gurnari, C. and Venditti, A. [2019], 'The emerging role of measurable residual disease detection in AML in morphologic remission', *Semin Hematol* **56**(2), 125–130.

[8] Busch, K., Klapproth, K., Barile, M., Flossdorf, M., Holland-Letz, T., Schlenner, S., Reth, M., Hofer, T. and HR., R. [2015], 'Fundamental properties of unperturbed haematopoiesis from stem cells in vivo.', *Nature* **518**(7540), 542–546.

[9] Cashman, J., Clark-Lewis, I., Eaves, A. and Eaves, C. [2002], 'Stromal-derived factor 1 inhibits the cycling of very primitive human hematopoietic cells in vitro and in NOD/SCID mice', *Blood* **99**(3), 792–799.

[10] Catlin, S. N., Busque, L., Gale, R. E., Guttorp, P. and Abkowitz, J. L. [2011], 'The replication rate of human hematopoietic stem cells in vivo', *Blood* **117**(17), 4460–4466.

[11] Colijn, C. and Mackey, M. C. [2005], 'A mathematical model of hematopoiesis—I. Periodic chronic myelogenous leukemia', *J Theor Biol* **237**(2), 117–132.

[12] Daldrup-Link, H., Henning, T. and Link, T. [2007], 'MR imaging of therapy-induced changes of bone marrow.', *Eur Radiol* **17**(3), 743–61.

[13] Desai, P., Mencia-Trinchant, N., Savenkov, O., Simon, M., Cheang, G., Lee, S., Samuel, M., Ritchie, E., Guzman, M., Ballman, K., Roboz, G. and Hassane, D. [2018], 'Somatic mutations precede acute myeloid leukemia years before diagnosis.', *Nat Med.* **24**(7), 1015–1023.

[14] Dircio-Maldonado, R., Flores-Guzman, P., Corral-Navarro, J., Mondragon-Garcia, I., Hidalgo-Miranda, A., Beltran-Anaya, F., Cedro-Tanda, A., Arriaga-Pizano, L., Balvanera-Ortiz, O. and Mayani, H. [2018], ' Functional Integrity and Gene Expression Profiles of Human Cord Blood-Derived Hematopoietic Stem and Progenitor Cells Generated In Vitro.', *Stem Cells Transl Med* **7**(8), 602–614.

[15] Donmez, A., Ozsan, F., Arik, B., Ozkayin, N., Cagirgan, S., Mir, S., Vural, F. and M., T. [2013], 'The clearance time of infused hematopoietic stem cell from the blood circulation.', *Transfus Apher Sci* **48**(2), 235–9.

[16] Fernández-García, M., Luisa Lamana, M., Hernando-Rodríguez, M., Sánchez-Domínguez, R., Bueren, J. and Yañez, R. [2018], 'Improved Hematopoietic Gene Therapy in a Mouse Model of Fanconi Anemia Mediated by Mesenchymal Stromal Cells', *Hum Gene Ther* **29**(3), 327–336.

[17] Hopman, R. and DiPersio, J. [2014], 'Advances in stem cell mobilization.', *Blood Rev* **28**(1), 31–40.

[18] Ishikawa, F., Yoshida, S., Saito, Y., Hijikata, A., Kitamura, H., Tanaka, S., Nakamura, R., Tanaka, T., Tomiyama, H., Saito, N., Fukata, M., Miyamoto, T., Lyons, B., Ohshima, K., Uchida, N., Taniguchi, S., Ohara, O., Akashi, K., Harada, M. and Shultz, L. D. [2007], 'Chemotherapy-resistant human AML stem cells home to and engraft within the bone-marrow endosteal region', *Nat Biotechnol* **25**(11), 1315.

[19] Karpova, D. and Bonig, H. [2015], 'Concise Review: CXCR4/CXCL12 Signaling in Immature Hematopoiesis-Lessons From Pharmacological and Genetic Models', *Stem Cells* **33**(8), 2391–2399.

[20] Kumar, S. and Geiger, H. [2017], 'HSC Niche Biology and HSC Expansion Ex Vivo', *Trends Mol Med* **23**(9), 799–819.

[21] Kunisaki, Y., Bruns, I., Scheiermann, C., Ahmed, J., Pinho, S., Zhang, D., Mizoguchi, T., Wei, Q., Lucas, D., Ito, K., Mar, J. C., Bergman, A. and Frenette, P. S. [2013], 'Arteriolar niches maintain haematopoietic stem cell quiescence', *Nature* **502**(7473), 637–643.

[22] Landau, H., Wood, K., Chung, D. J., Koehne, G., Lendvai, N., Hassoun, H., Lesokhin, A., Hoover, E., Zheng, J., Devlin, S. M. and Giralt, S. [2016], 'Fractionated stem cell infusions for patients with plasma cell myeloma undergoing autologous hematopoietic cell transplantation', *Leuk Lymphoma* **57**(8), 1781–1785.

[23] Lee-Six, H., Obro, N., Shepherd, M., Grossmann, S., Dawson, K., Belmonte, M., Osborne, R., Huntly, B., Martincorena, I., Anderson, E., O'Neill, L., Stratton, M., Laurenti, E., Green, A., Kent, D. and Campbell, P. [2018], 'Population dynamics of normal human blood inferred from somatic mutations.', *Nature* **561**(7724), 473–478.

[24] Marciniak-Czochra, A., Stiehl, T., Ho, A. D., Jager, W. and Wagner, W. [2009], 'Modeling of asymmetric cell division in hematopoietic stem cells–regulation of self-renewal is essential for efficient repopulation', *Stem Cells Dev* **18**(3), 377.

[25] Mayle, A., Luo, M., Jeong, M. and Goodell, M. [2013], 'Flow cytometry analysis of murine hematopoietic stem cells.', *Cytometry A* **83**(1), 27–37.

[26] Mead, A. J. and Mullally, A. [2017], 'Myeloproliferative neoplasm stem cells', *Blood* **129**(12), 1607–1616.

[27] Morgan, R. A., Gray, D., Lomova, A. and Kohn, D. B. [2017], 'Hematopoietic Stem Cell Gene Therapy: Progress and Lessons Learned', *Cell Stem Cell* **21**(5), 574–590.

[28] Nie, Y., Han, Y.-C. and Zou, Y.-R. [2008], 'CXCR4 is required for the quiescence of primitive hematopoietic cells', *J Exp Med* **205**(4), 777–783.

[29] Nilsson, S. K., Johnston, H. M., Whitty, G. A., Williams, B., Webb, R. J., Denhardt, D. T., Bertoncello, I., Bendall, L. J., Simmons, P. J. and Haylock, D. N. [2005], 'Osteopontin, a key component of the hematopoietic stem cell niche and regulator of primitive hematopoietic progenitor cells', *Blood* **106**(4), 1232–1239.

[30] Pantin, J., Purev, E., Tian, X., Cook, L., Donohue-Jerussi, T., Cho, E., Reger, R., Hsieh, M., Khuu, H., Calandra, G., Geller, N. L. and Childs, R. [2017], 'Effect of high-dose plerixafor on CD34+ cell mobilization in healthy stem cell donors: results of a randomized crossover trial.', *Haematologica* **102**(3), 600–609.

[31] Pedersen, R., Andersen, M., Stiehl, T. and JT, O. [2021], 'Mathematical modelling of the hematopoietic stem cell-niche system: Clonal dominance based on stem cell fitness ', *J Theor Biol* **518**, 110620.

[32] Schroeder, M. A. and DiPersio, J. F. [2012], 'Mobilization of hematopoietic stem and leukemia cells', *J Leukoc Biol* **91**(1), 47–57.

[33] Shen, Z.-H., Zeng, D.-F., Kong, P.-Y., Ma, Y.-Y. and Zhang, X. [2016], 'AMD3100 and G-CSF disrupt the cross-talk between leukemia cells and the endosteal niche and enhance their sensitivity to chemotherapeutic drugs in biomimetic polystyrene scaffolds', *Blood Cells Mol Dis* **59**, 16–24.

[34] Shen, Z.-H., Zeng, D.-F., Ma, Y.-y., Zhang, X., Zhang, C. and Kong, P.-Y. [2015], 'Are there any new insights for G-CSF and/or AMD3100 in chemotherapy of haematological malignants?', *Med Oncol* **32**(12), 262.

[35] Shi, P., Miller, L. and Isola, L. [2014], 'Prospective study of mobilization kinetics up to 18 hours after late-afternoon dosing of plerixafor.', *Transfusion* **54**(5), 1263–1268.

[36] Spivak, J. L. [2017], 'Myeloproliferative Neoplasms', *N Engl J Med* **376**(22), 2168–2181.

[37] Stiehl, T., Ho, A. D. and Marciniak-Czochra, A. [2014], 'The impact of CD34+ cell dose on engraftment after SCTs: Personalized estimates based on mathematical modeling', *Bone Marrow Transplant* **49**(1), 30–37.

[38] Stiehl, T., Wang, W., Lutz, C. and Marciniak-Czochra, A. [2020], 'Mathematical modeling provides evidence for niche competition in human AML and serves as a tool to improve risk stratification', *Cancer Res.* **80**(18), 3983–3992.

[39] Sugiyama, T., Kohara, H., Noda, M. and Nagasawa, T. [2006], 'Maintenance of the Hematopoietic Stem Cell Pool by CXCL12-CXCR4 Chemokine Signaling in Bone Marrow Stromal Cell Niches', *Immunity* **25**(6), 977–988.

[40] Thiele, J., Laubert, A., Vykoupil, K. and Georgii, A. [1985], 'Autopsy and clinical findings in acute leukemia and chronic myeloproliferative diseases–an evaluation of 104 patients.', *Pathol Res Pract* **179**(3), 328–336.

[41] Vaidya, A. and Kale, V. [2015], 'Hematopoietic Stem Cells, Their Niche, and the Concept of Co-Culture Systems: A Critical Review.', *J Stem Cells* **10**(1), 13–31.

[42] Vandekerckhove, J. [2008], *General simulated annealing algorithm*, MATLAB Central File Exchange, Last accessed: 2020-05-15.
URL: *https://www.mathworks.com/matlabcentral/fileexchange/10548-general-simulated-annealing-algorithm*

[43] Vanegas, N. and Vernot, J. [2017], 'Loss of quiescence and self-renewal capacity of hematopoietic stem cell in an in vitro leukemic niche.', *Exp Hematol Oncol.* **6**, 2.

[44] Wang, W., Stiehl, T., Raffel, S., Hoang, V. T., Hoffmann, I., Poisa-Beiro, L., Saeed, B. R., Blume, R., Manta, L., Eckstein, V., Bochtler, T., Wuchter, P., Essers, M., Jauch, A., Trumpp, A., Marciniak-Czochra, A., Ho, A. D. and Lutz, C. [2017], 'Reduced hematopoietic stem cell frequency predicts outcome in acute myeloid leukemia', *Haematologica* **102**(9), 1567–1577.

[45] Watts, M. and Linch, D. [2016], 'Optimisation and quality control of cell processing for autologous stem cell transplantation.', *Br J Haematol* **175**(5), 771–783.

[46] Welschinger, R., Liedtke, F., Basnett, J., Dela Pena, A., Juarez, J. G., Bradstock, K. F. and Bendall, L. J. [2013], 'Plerixafor (AMD3100) induces prolonged mobilization of acute lymphoblastic leukemia cells and increases the proportion of cycling cells in the blood in mice', *Exp Hematol* **41**(3), 293–302.e1.

[47] Wright, D., Wagers, A., Gulati, A., Johnson, F. and Weissman, I. [2001], 'Physiological migration of hematopoietic stem and progenitor cells.', *Science* **294**(5548), 1933–1936.

[48] Zhang, J., Niu, C., Ye, L., Huang, H., He, X., Tong, W.-G., Ross, J., Haug, J., Johnson, T., Feng, J. Q., Harris, S., Wiedemann, L. M., Mishina, Y. and Li, L. [2003], 'Identification of the haematopoietic stem cell niche and control of the niche size', *Nature* **425**(6960), 836–841.

[49] Zhang, Y. and Gao, Y. [2016], 'Novel chemical attempts at ex vivo hematopoietic stem cell expansion', *Int J Hematol* **103**(5), 519–529.
